# Supplementary material for: Tumor immune microenvironment reconstitution in patient-derived organoids enables therapy modeling for NSCLC
Source: Cell Rep Methods. 2026 May 13;6(6):101339. doi: 10.1016/j.crmeth.2026.101339 (PMC13282660; doi:10.1016/j.crmeth.2026.101339)
Supplement: Document S1. Figures S1–S4 and Tables S1–S3 [file mmc1.pdf]

Cell Reports Methods, Volume *6*

Supplemental information

Tumor immune microenvironment reconstitution in patient-derived organoids enables therapy modeling for NSCLC

Enrique Podaza, Jared Capuano, Hui-Hsuan Kuo, Majd Al Assaad, Geoffrey Markowitz, M. Victoria Revuelta, John Nguyen, Adriana Irizarry, Hiranmayi Ravichandran, Sarah Ackermann, Troy Kane, Jyothi Manohar, Alyssa Duren-Lubanski, Michael Sigouros, Jenna Moyer, Bhavneet Bhinder, Pooja Chandra, Murtaza Malbari, Karsten Boehnke, Juan Miguel Mosquera, Vivek Mittal, Andrea Sboner, Hamza Gokozan, Nasser Altorki, Olivier Elemento, and M. Laura Martin

Supplemental information

Tumor immune microenvironment reconstitution in patient-derived organoids enables therapy modeling for NSCLC

Enrique Podaza, Jared Capuano, Hui-Hsuan Kuo, Majd Al Assaad, Geoffrey Markowitz, M. Victoria Revuelta, John Nguyen, Adriana Irizarry, Hiranmayi Ravichandran, Sarah Ackermann, Troy Kane, Jyothi Manohar, Alyssa Duren-Lubanski, Michael Sigouros, Jenna Moyer, Bhavneet Bhinder, Pooja Chandra, Murtaza Malbari, Karsten Boehnke, Juan Miguel Mosquera, Vivek Mittal, Andrea Sboner, Hamza Gokozan, Nasser Altorki, Olivier Elemento and M. Laura Martin

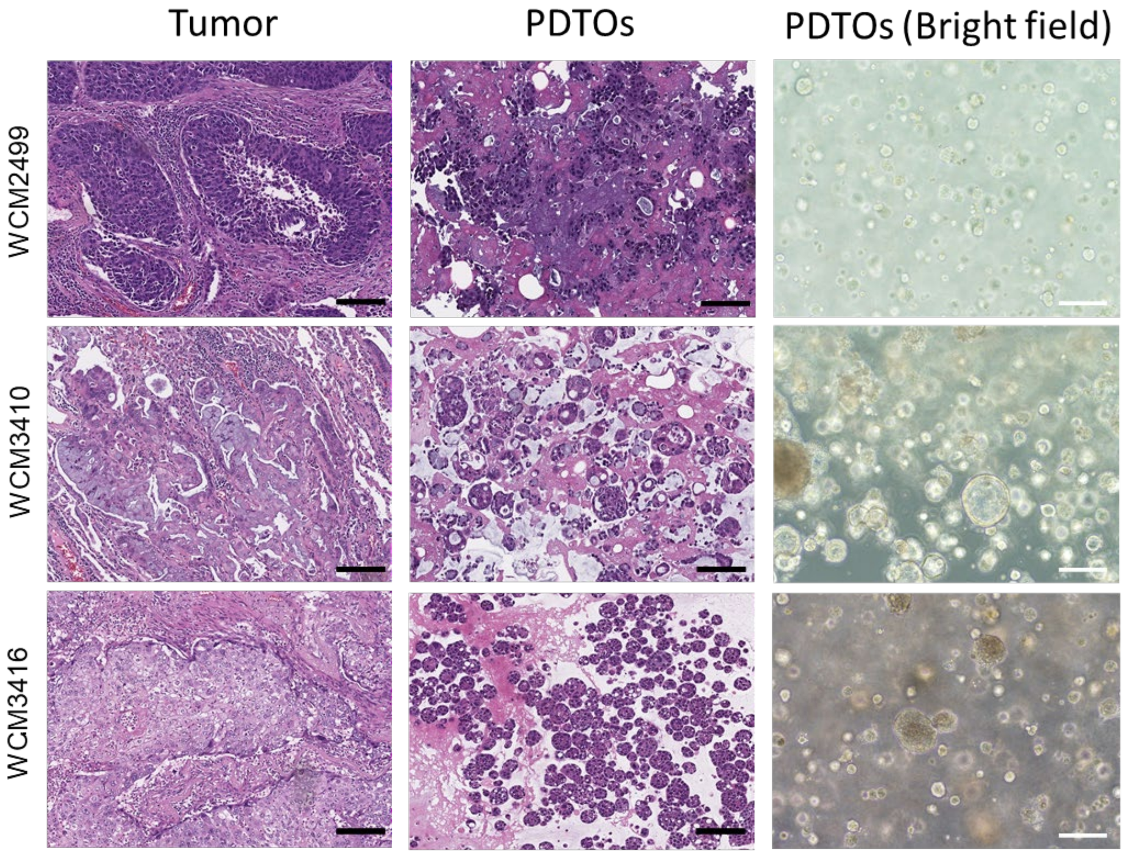

Figure S1. H&E of tumor and PDTO pairs for 3 cases of different subtypes of NSCLC: LCNEC (WCM2499), LUAD (WCM3410) and LUSC (WCM3416). Bright field images for the PDTOs are also displayed. Bars indicate 100µm. Related to Figure 1.

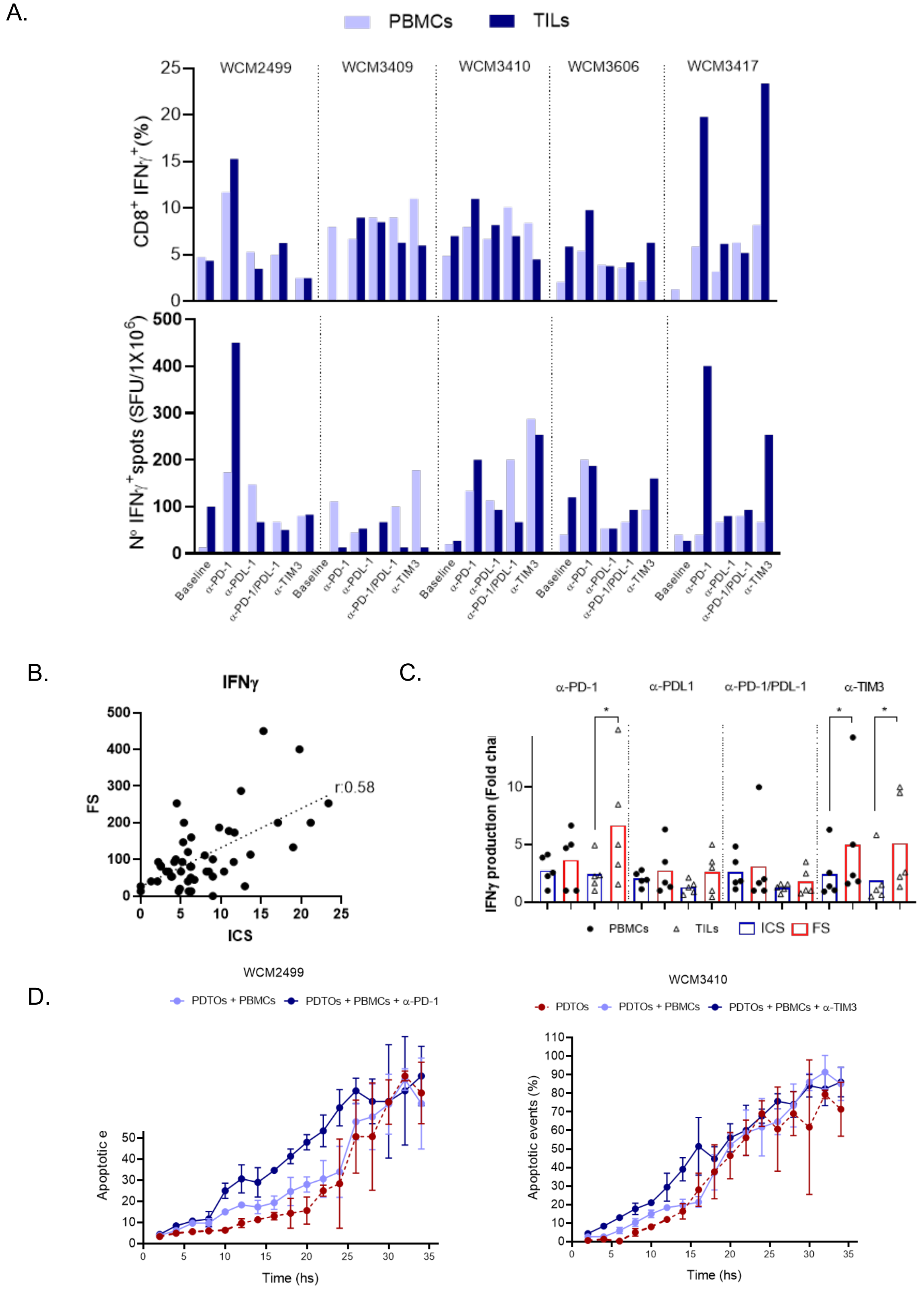

A.
PBMCs
TILs
WCM2499
WCM3409
WCM3410
WCM3606
WCM3417
CD8+ IFNγ+(%)
Nº IFNγ+spots (SFU/1X10^6)
Baseline
α-PD-1
α-PDL-1
α-PD-1/PDL-1
α-TIM3
B.
IFNγ
FS
ICS
r:0.58
C.
α-PD-1
α-PDL1
α-PD-1/PDL-1
α-TIM3
IFNγ production (Fold cha
PBMCs
TILs
ICS
FS
D.
WCM2499
PDTOs + PBMCs
PDTOs + PBMCs + α-PD-1
Apoptotic e
Time (hs)
WCM3410
PDTOs
PDTOs + PBMCs
PDTOs + PBMCs + α-TIM3
Apoptotic events (%)
Time (hs)

Figure S2. Optimization of functional assays for the evaluation of T-cells IFNγ secretion and its modulation by immune-checkpoint inhibitors after co-culture with PDTOs.

A. INFγ secretion recorded by intracellular staining (ICS) and fluorospot (FS). Bars graphs display the frequencies of T-cells producing IFNγ, baseline and upon the addition of different mAbs. Frequencies are shown as percentage of $CD8^+$ $IFN\gamma^+$ for ICS (Upper graph) and as spots forming units (SFU) per 1 x 10^6 $CD8^+$ T-cells for FS (Lower graph). PBMCs and TILs frequencies for each patient are shown (n=5). **B.** Cross assay validation analysis. Correlation between IFNγ measures recorded by ICS and FS. Statistical significance was determined by Spearman's correlation (two tails, CI=95%). Spearman r coefficients are shown for each cytokine **C.** Comparison of IFNγ production by T-cells (TILs and PBMCs) in the presence of the different mAbs (α-PD-1, α-PDL1, α-PD-1/PDL1 and α-TIM3) recorded by ICS and FS. Values are shown as fold change relative to the control levels. (*) Statistical significance was calculated by Kruskal-wallis test, Dunn's multiple comparisons post-test (p=0.05). Black dots: PBMCs, White triangles: TILs, Blue bars: ICS, and Red bars: FS. n=5. **D.** Extended tumor killing assays**.** In this panel we show representative killing curves for two patients recorded up to 35 h. The curves show higher levels of dispersion at longer culture times. For this reason, 12 h of culture is shown on the main figures for all patients. Related to Figure 2.

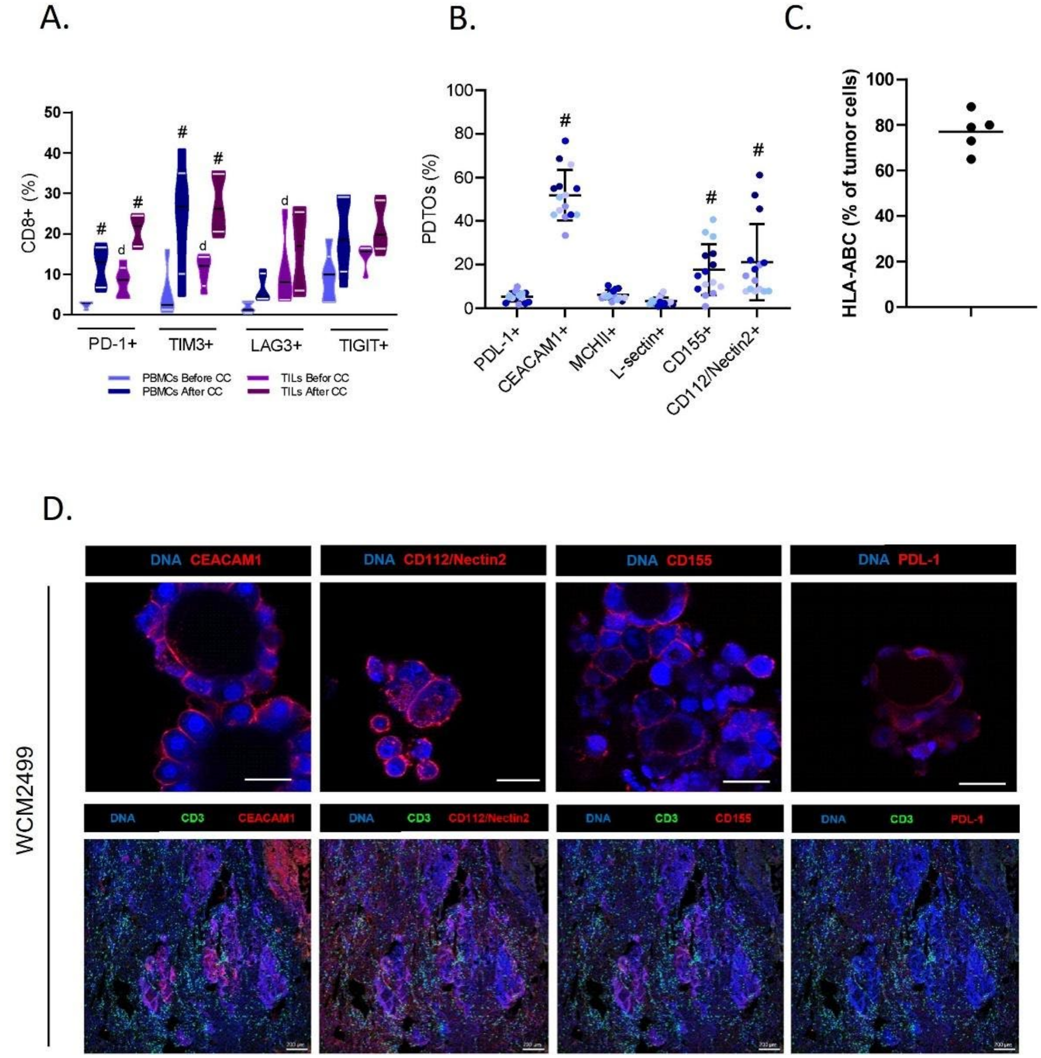

Figure S3. **Immune-checkpoints expression on T-cells and paired PDTOs**. **A.** Expression of inhibitory receptors Percentage of CD8+ cells expressing the inhibitory receptors PD-1, TIM3, LAG3 and TIGIT at day 0 and 14. Violin plots displaying the mean (black line) and quartiles (gray lines) for each inhibitory receptor in PBMCs and TILs before and after co-culture. # Indicates different than the condition before co-culture. d indicates significantly different than the PBMCs before co-culture. Differences were determined by Kruskal-Wallis test and Dunn's multiple comparison post-test (p=0.05) **B.** Percentage of PDTOs expressing PDL1, TIM3 ligand CEACAM, TIGIT ligands CD112/Nectin2, CD155, LAG3 ligands MCHII, and LSECtin. PDTO lines were stained 3 times at different passages between passage 6 and 15. # Indicate significantly higher than the PDL1 expression. Differences were determined by Kruskal-Wallis test and Dunn's multiple comparison post-test (p=0.05) **C.** Assessment of HLA-ABC expression on PDTOs before co-culture. Shown are the percentages of PDTOs positive for HLA-ABC**. D.** Representative confocal microscopy images of WCM2499 showing the surface expression of PDL1, CEACAM1, CD112/Nectin2, and CD155. Blue: DNA (DAPI); red: Immune checkpoint ligands (upper row). Imaging Cytoff of a tumor resection of WCM2499 showing the overall lymphocytic infiltration (CD3, green), DNA (blue) and expression of PDL1, CEACAM, CD112/Nectin2 and CD155 (red). Scale bars: 200µm (lower row). Related to Figure 3.

A.

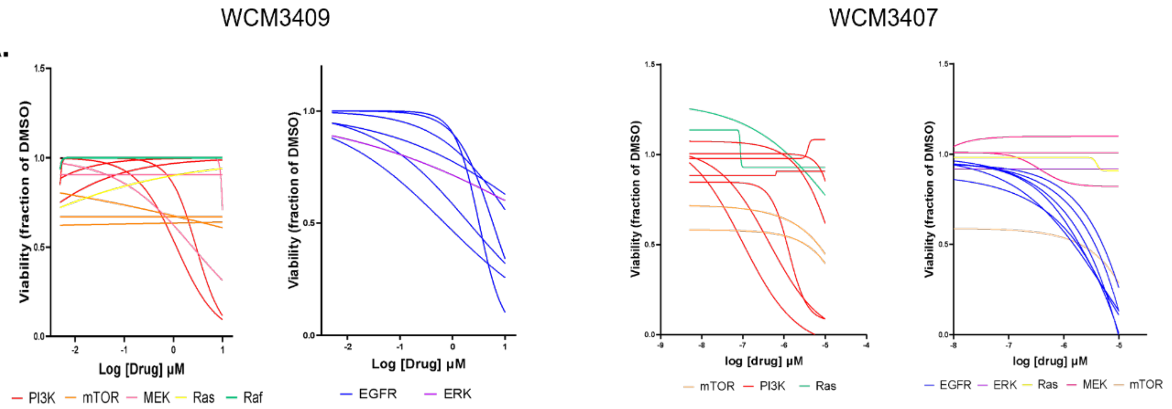

B.

WCM3409

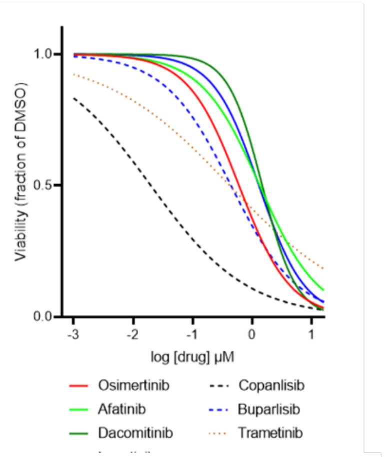

	EC10	EC20	EC25	EC50
**Afatinib**	0.268	0.5985	0.800	2.394
**Lapatinib**	0.431	0.47	0.9	1.877
**Osimertinib**	0.064	0.18	0.216	0.726
**Dacomitinib**	0.03	0.5	1.1	2.0
**Copanlisib**	0.005	0.016	0.021	0.064
**Buparlisib**	0.008	0.017	0.398	0.7655
**Trametinib**	0.015	0.032	0.08	0.136

C.

WCM3407

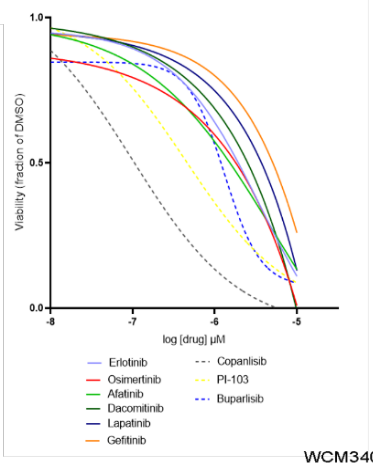

	EC10	EC20	EC25	EC50
**Afatinib**	5.85×10^{-7}	1.32×10^{-6}	1.32×10^{-6}	5.3×10^{-6}
**Lapatinib**	0.044	0.09	0.132	0.398
**Osimertinib**	4.13	9.3	12.4	37.20
**Dacomitinib**	0.07	0.162	0.216	0.6485
**Erlotinib**	5.85×10^{-7}	1.32×10^{-6}	1.32×10^{-6}	5.3×10^{-6}
**Gefitinib**	1.163×10^{-5}	2.62×10^{-5}	3.49×10^{-5}	0.0001047
**Copanlisib**	1.09×10^{-8}	2.46×10^{-8}	3.28×10^{-8}	9.86×10^{-8}
**Buparlisib**	1.47×10^{-7}	3.29×10^{-7}	4.39×10^{-7}	1.32×10^{-6}
**PI-103**	4.94×10^{-8}	1.11×10^{-7}	1.48×10^{-7}	4.44×10^{-7}

D.

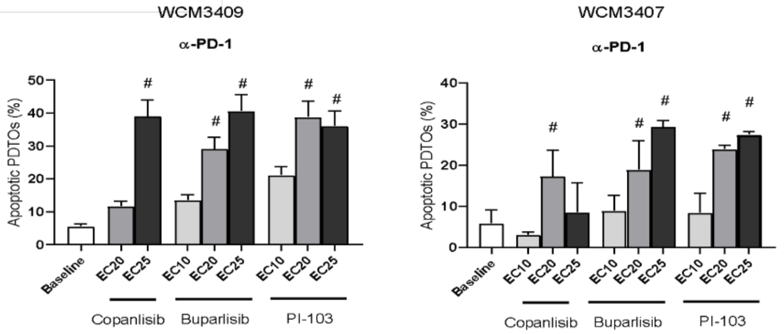

Figure S4. Kras G12A NSCLC-PDTOs sensitivity to target inhibitors.

PDTOs were digested into a single cell suspension and cells were plated in a 384 well plate at a density of 1000 cells per well in 8ul droplets (1:2 media:Matrigel). Plates were centrifuged briefly to ensure the cells were at the bottom of the well and 15μl of media were added. Cells were incubated for 72h to allow the cells to form PDTOs and afterwards the drugs were added and incubated for additional 96h. Concentrations ranging between 10 and 0 (serial 1/3 dilutions) were assessed for 23 different inhibitors against: EGFR (Lapatinib, Osimertinib, Dacomitinib, Afatinib, Erlotinib and Gefitinib), PI3K (Idelalisib, Copanlisib, PI-103, Buparlisib, GSK2636771, Parsaclisib and Erganelisib), mTOR (Temsirolimus, Rapamycin and Everolimus), ERK (Ulixertinib), Raf (AZ628 and Dabrafenib), Ras (AMF510) and MEK (Binimetinib,Trametinib and Selumetinib). The readout was performed using CellTiterGlo®3D reagent according to the manufacturer's protocol. Luminescence was measured by the Biotek Synergy H4 plate reader. Full dose-response curves are depicted for each patient in **A**. Each color represents a particular target. Dose response curves for the hit compounds and their estimated effective concentrations 10, 20, 25 and 50 are depicted for WCM3409 (**B**) and for WCM3407 (**C**). **D**. Apoptotic PDTOs (%) recorded for cells treated with α-PD-1 and PI3K inhibitors (Copanlisib, Buparlisib and PI-103). Mean + SD is displayed. The conditions depicted are those were the fold change between the ICI vs ICI + target inhibitor was greater than 2 and the observations across duplicates were consistent. Differences were determined by Kruskal-Wallis test and Dunn's multiple comparison post-test (p=0.05). # means significantly different than baseline. Related to Figure 3.

A.

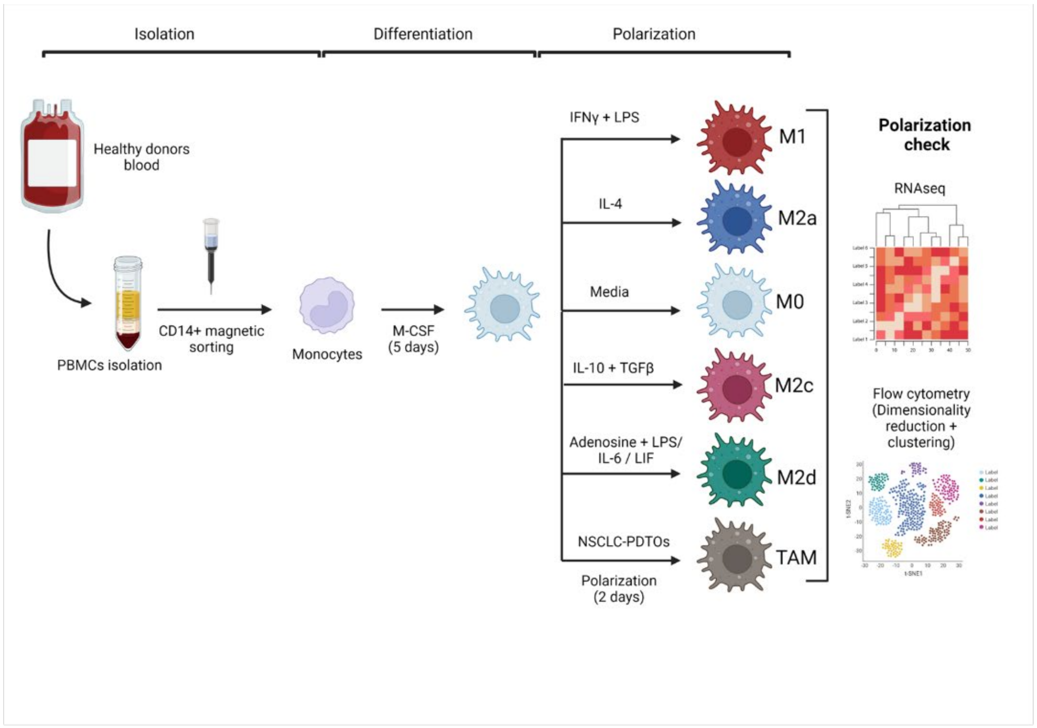

B.

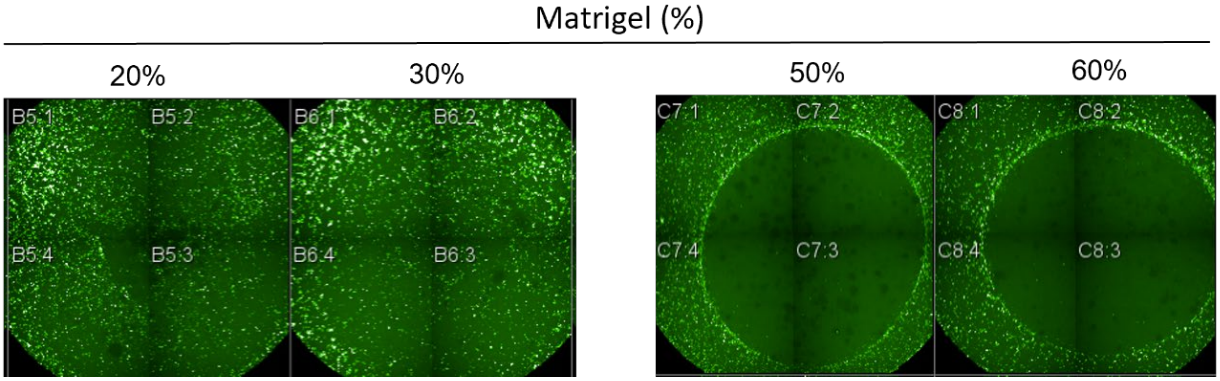

Figure S5. Macrophage generation and characterization workflow.

A. Macrophage differentiation and polarization protocol. FBMCs were isolated from healthy donor leukopacks. Monocytes were purifed from PBMCs suspensions by magneto sorting (CD14 positive selecton). Purifed monocytes were differentiated into macrophages by culturing them in the presence of M-CS during 5 days. MO macrophages were polarized to the different subsets by culturing them during 48hs with subset specifc factors. For the particular case of TAMs polarization, MO macrophages were oo cultured with tumor oells in a 1:10 ratio (MO: tumor oell). After oo-culture, CD45+ magne to sorting was used to eliminate tumor oells. Polarized macrophages were characterized by RNAseq (followed by qPCR validation of selected genes) and flow cytometry employing conventional M1/M2 markers as well as Lung-associated macrophages markers. B. Matrigel concentration assessment. CFSE-stained macrophages were co-culture with PDTOs employing different Matrigel concentrations (20, 30, 50 and 80%) and images were acquired at day 12 of culture. Related to Figure 4.

ID	TILs
WCM1327	Fail
WCM1335	Fail
WCM1332	Fail
WCM1341	Fail
WCM1518	Succeed
WCM1532	Fail
WCM1536	Succeed
WCM1548	Succeed
WCM1547	Fail
WCM1471	Fail
WCM1478	Fail
WCM1442	Fail
WCM1412	Fail
WCM1445	Fail

Table S1.TILs expansion outcome for tumor samples digested with collagenase IV. Related to Figure 1.

Patient	Gender	Race/ Ethnicity	Smoking status	Cancer stage	Lesion size on pathologic examination (cm)	T stage	N stage	M stage
**WCM3407**	M	White	Former	IIB	4.0	T3	N0	M0
**WCM2499**	F	White	Former	IIIA	7,6	T4	N0	M0
**WCM3606**	F	African American	Former	IIA	3,8	T2a	N1	M0
**WCM3417**	F	White	Never	IA3	3	T1c	N0	M0
**WCM3409**	F	African American	Never	IA3	2,9	T1c	N0	M0
**WCM3410**	F	White	Former	IIIA	7,3	T4	N0	M0
**WCM3602**	M	White	Current	IIIA	3,3	T2a	N2	M0
**WCM3603**	F	White	Never	IA2	1,7	T1b	N0	M0
**WCM3604**	M	White	Never	IB	3,3	T2a	N0	M0
**WCM3605**	F	White	Never	IA3	2,6	T1c	N0	M0
**WCM3413**	M	White	Former	IIIA	3	T1b	N0	M0
**WCM3066**	M	White	Current	IA2	1,9	T3	N1	M1a
**WCM3607**	F	White	Never	IA3	2,1	T1c	N0	M0
**WCM3416**	M	White	Former	IIB	5,5	T3	N0	M0
**WCM3608**	M	Asian	Never	IB	3,5	T2a	N0	M0
**WCM3083**	F	unknown	Current	IVA	4	T3	N1	M1a
**WCM3289**	M	Asian	Former	IIIB	6,2	T3	N2	M0

Table S2. Patients clinical information. Related to Figure 1

ID	TILs	Histopathological review
WCM3407	No	Scattered infiltration. Presence of macrophages
WCM2499	Yes	High lymphocytic infiltration in tumor stroma
WCM3606	Yes	No infiltration. T-cells present in surrounding normal tissue
WCM3417	Yes	No infiltration. T-cells present in surrounding normal tissue
WCM3409	Yes	Moderate and scattered infiltration
WCM3410	Yes	High Lymphocytic infiltration in tumor stroma
WCM3413	No	Scattered infiltration
WCM3416	No	Scattered infiltration. Presence of macrophages
WCM3289	Yes	No infiltration. T-cells present in surrounding normal tissue

Table S3- Tumor immune infiltration histopathological report and TILs culture success (Yes-Succeed, no: Fail).
Related to Figure 1.
