# Supplementary material for: Tumor immune microenvironment reconstitution in patient-derived organoids enables therapy modeling for NSCLC
Source: Cell Rep Methods. 2026 May 13;6(6):101339. doi: 10.1016/j.crmeth.2026.101339 (PMC13282660; doi:10.1016/j.crmeth.2026.101339)
Supplement: Document S2. Article plus supplemental information [file mmc3.pdf]

Cell Reports Methods

Article

Tumor immune microenvironment reconstitution in patient-derived organoids enables therapy modeling for NSCLC

Graphical abstract

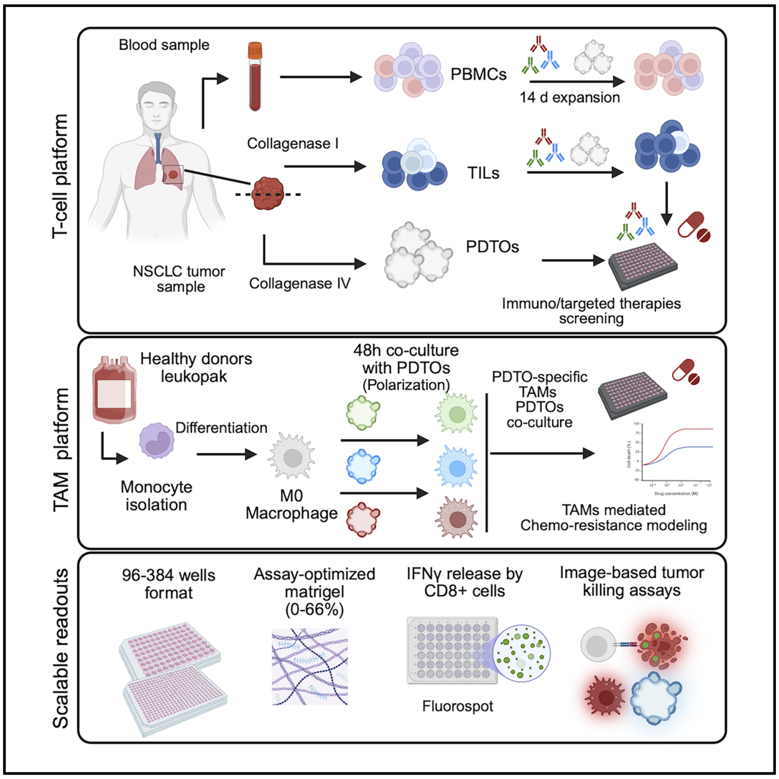

Authors

Enrique Podaza, Jared Capuano, Hui-Hsuan Kuo, ..., Nasser Altorki, Olivier Elemento, M. Laura Martin

Correspondence

mlm4001@med.cornell.edu

In brief

Podaza et al. develop scalable strategies for reconstituting tumor immune microenvironment components in patient-derived organoid cultures. Their platforms enable high-throughput screening of immune checkpoint inhibitor combinations and reveal how tumor-associated macrophages influence chemotherapy sensitivity, providing practical tools for modeling immunotherapy responses and identifying effective combination therapies in non-small cell lung cancer.

Highlights

- Concurrent tumor and lymphocyte isolation from patient samples
- Scalable assays for individual responses to checkpoint inhibitor and targeted therapies
- PDTO co-culture generates tumor-specific macrophages without exogenous polarization
- High-throughput TIME-PDTO platforms model immunotherapy and chemoresistance

Podaza et al., 2026, Cell Reports Methods 6, 101339
June 15, 2026 © 2026 The Author(s). Published by Elsevier Inc.
https://doi.org/10.1016/j.crmeth.2026.101339

CellPress

Cell Reports Methods

CellPress
OPEN ACCESS

Article

Tumor immune microenvironment reconstitution in patient-derived organoids enables therapy modeling for NSCLC

Enrique Podaza,[1,6] Jared Capuano,[1] Hui-Hsuan Kuo,[1] Majd Al Assaad,[1,3] Geoffrey Markowitz,[2] M. Victoria Revuelta,[4] John Nguyen,[1] Adriana Irizarry,[1] Hiranmayi Ravichandran,[1] Sarah Ackermann,[1] Troy Kane,[1] Jyothi Manohar,[1] Alyssa Duren-Lubanski,[1] Michael Sigouros,[1] Jenna Moyer,[1] Bhavneet Bhinder,[1,7] Pooja Chandra,[1,7] Murtaza Malbari,[2] Karsten Boehnke,[5] Juan Miguel Mosquera,[1,3] Vivek Mittal,[2] Andrea Sboner,[1,3,7] Hamza Gokozan,[3] Nasser Altorki,[2] Olivier Elemento,[1,6,7,8] and M. Laura Martin[1,8,9,*]

[1]Caryl and Israel Englander Institute for Precision Medicine, Weill Cornell Medicine, New York, NY 10021, USA
[2]Cardiothoracic Surgery, Weill Cornell Medical College, New York, NY 10021, USA
[3]Department of Pathology and Laboratory Medicine, Weill Cornell Medical College, New York, NY 10021, USA
[4]Department of Hematology and Oncology, Weill Cornell Medical College, New York, NY 10021, USA
[5]Eli Lilly and Company, Lilly Oncology, Discovery Technologies, New York, NY 10016, USA
[6]Department of Physiology and Biophysics, Weill Cornell Medicine, New York, NY 10021, USA
[7]Institute for Computational Biomedicine, Weill Cornell Medicine, New York, NY 10021, USA
[8]These authors contributed equally
[9]Lead contact
*Correspondence: mlm4001@med.cornell.edu
https://doi.org/10.1016/j.crmeth.2026.101339

MOTIVATION Patient-derived tumor organoids have emerged as powerful tools for personalized medicine and drug screening, yet their application to immunotherapy remains limited by the absence of tumor immune microenvironment (TIME) components. Existing co-culture approaches are predominantly low-throughput and lack standardized protocols for reconstituting key immune populations, such as tumor-infiltrating lymphocytes and tumor-associated macrophages. This methodological gap prevents scalable assessment of combination immunotherapies and limits our ability to model how TIME components influence therapeutic responses. To address these limitations, we developed reproducible, high-throughput strategies for TIME reconstitution in organoid cultures, enabling functional screening of immune checkpoint inhibitor combinations, sequential therapies, and TIME-mediated treatment resistance, using non-small cell lung cancer as a model system.

SUMMARY

Non-small cell lung cancer (NSCLC) remains a leading cause of cancer-related mortality. Despite various therapeutic options, treatment resistance is common, underscoring the need for effective combination therapies and reliable pre-clinical models for patient-specific evaluation. Here, we describe strategies for reconstituting tumor immune microenvironment (TIME) components within patient-derived tumor organoid (PDTO) cultures. We established a tumor processing pipeline that enables concurrent expansion of tumor-infiltrating lymphocytes (TILs) and PDTO generation from the same resection. We optimized scalable assays to assess IFN-γ secretion and T cell cytotoxicity with immune checkpoint inhibitors (alone or in combination) and targeted inhibitors, capturing inter-patient heterogeneity and intra-patient variations between TILs and peripheral blood mononuclear cells (PBMCs). Additionally, we developed methods for differentiating PDTO-specific tumor-associated macrophages (TAMs) and established PDTO-TAM co-culture systems to evaluate TAM effects on PDTO growth and chemotherapy sensitivity. All approaches are scalable to high-throughput levels, highlighting the value of TIME-PDTO co-cultures for therapeutic modeling and precision medicine.

© 2026 The Author(s). Published by Elsevier Inc.
This is an open access article under the CC BY license (http://creativecommons.org/licenses/by/4.0/).

INTRODUCTION

Non-small cell lung cancer (NSCLC) is a leading cause of cancer-related deaths, with an incidence rate of 11.4%.[1] Treatment is primarily determined by disease stage, ranging from surgical resection and local radiotherapy to adjuvant platinum-based chemotherapy and concurrent chemo-radiotherapy.[2,3] Treatment decisions are also guided by specific biomarkers. Patients with mutations in EGFR, ALK, ROS1, or KRAS are eligible for targeted therapies, while PD-L1 expression and tumor-infiltrating lymphocytes (TILs) inform the use of immune checkpoint inhibitors (ICIs).[4,5] For patients lacking specific biomarkers or with advanced disease stages, combination therapies are being explored to target multiple pathways and improve treatment efficacy. These strategies, such as combining chemotherapy with immunotherapy or targeted therapies, aim to enhance therapeutic outcomes and overcome resistance, a frequent phenomenon among patients with NSCLC treated with monotherapies.[6–9] Therapy resistance can emerge from tumor-intrinsic mechanisms or from the tumor immune microenvironment (TIME).[10–12] Factors such as the quantity and phenotype of TILs influence the effectiveness of ICIs,[13,14] while the presence and spatial distribution of tumor-associated macrophages (TAMs) can hinder therapeutic effectiveness.[11,15,16]

In the quest for more effective therapies, patient-derived tumor organoids (PDTOs) have emerged as powerful tools for disease modeling, drug screening, and personalized medicine.[17–19] PDTOs retain critical mutational and structural features of the original tumor, making them an invaluable *in vitro* model for predicting clinical responses. PDTOs have been particularly successful in evaluating responses to chemotherapy and targeted therapies, providing a reliable platform for personalized treatment planning.[20–22] However, the potential of PDTOs for immunotherapy screening remains largely underexplored. Most studies have focused on low-throughput assessments, primarily investigating responses to anti-PD-1 inhibitors.[23–25] There is a pressing need to expand these models for high-throughput immunotherapy screens, particularly to better understand immune responses and to identify novel combination therapies that could improve patient outcomes. Developing well-defined strategies for TIME reconstitution in PDTOs and evaluating different immune-cell sources will be key to advancing their value for immunotherapy research and to better represent its influence on therapy outcomes.

To address this, we have optimized a tumor processing protocol that enables PDTO establishment and TIL isolation from the same lung tumor resections. We also report scalable functional assays to evaluate T cell effector functions by testing various combinations of ICIs and targeted inhibitors and by comparing autologous TILs and peripheral blood mononuclear cells (PBMCs) from the same patient. Furthermore, we optimized a reprogramming strategy for the reconstitution of patient-specific TAMs, and in proof-of-concept experiments, we demonstrated their effect on PDTO growth and sensitivity to treatment. Our results emphasize the immense potential of PDTO-TIME co-culture (with T-cells or TAMs) as highly promising preclinical models for therapeutic screening.

RESULTS

Protocol optimization enables NSCLC-PDTO establishment and TIL isolation from the same tumor sample

To create PDTO-T cell co-cultures, we optimized a hybrid protocol in which tumor samples are split in half: one-half is digested with collagenase IV for PDTO development and the other with collagenase I for isolation of TILs (Figure 1A). Initial attempts to grow both types of cells using only one type of collagenase for tumor digestion failed to yield successful cultures of either PDTOs (with collagenase I) or TILs (with collagenase IV) (Table S1). Peripheral blood is collected as part of the surgical procedure and immediately processed for PBMC isolation and cryopreservation.

From November 2020 until June 2022, we collected 17 tumor resections from patients with NSCLC who were treatment-naïve. Patients' clinical data is depicted in Table S2. The tumors consisted mostly of invasive adenocarcinomas (LUAD, n = 15) and included 1 squamous cell carcinoma (LUSC) and 1 large cell neuroendocrine carcinoma (LCNEC). PDTOs were successfully established for 9/17 (52.9%) cases: 7 LUAD, 1 LUSC, and 1 LCNEC. TILs were isolated and expanded for 7/17 (41.17%) cases. PDTO generation success rate was positively associated with tumor size but not with tumor stage or histological features (Figure 1B; Table 1). However, although a trend was observed between TIL success rate and tumor size or stage, these associations were not statistically significant (Figure 1B). Additionally, TIL success rate was not associated with the overall level of tumor lymphocytic infiltration (Table S3). Altogether, these results demonstrate the feasibility of isolating and culturing matched PDTOs and TILs from the same tumor.

Histopathologic and morphological review comparing PDTOs and their originating tumors revealed that the LUSC (WCM3416) and LCNEC (WCM2499)-derived PDTOs retained the structure and cell composition of their tumor of origin (Figure S1). Surprisingly, LUAD-derived PDTOs were predominantly acinar and/or mucinous (WCM3409 is shown as an illustrative LUAD case), whereas their originating tumor patterns were mostly micropapillary, suggesting that the culture condition may promote a change in morphology. Additionally, we determined the genomic concordance between tumor and PDTO pairs for point mutations, indels, and copy number alterations. The initial analysis of 19 driver genes in lung cancer, previously reported by TCGA, showed that genomic alterations in TP53 and KRAS were the most frequent (33% in our cohort vs. 46%–33%, respectively, in TCGA), KRAS and EGFR alterations were mutually exclusive, and deletions in CDKN2A were highly frequent (50%) (Figure 1C). Overall, most driver alterations were preserved in PDTOs.

Whole-exome sequencing (WES) data, available for 3 of the 5 cases evaluated in the co-cultures, enabled us to analyze the concordance of predicted neoantigenic mutations between tumors and PDTOs. The overall concordance was >80% for WCM3410 (88%) and WCM2499 (82%), while it was 68% for WCM3409 (Figure 1D). In the latter case, the PDO presented a larger number of mutations that were not present in the tumor (Figure 1E).

CellPress
OPEN ACCESS

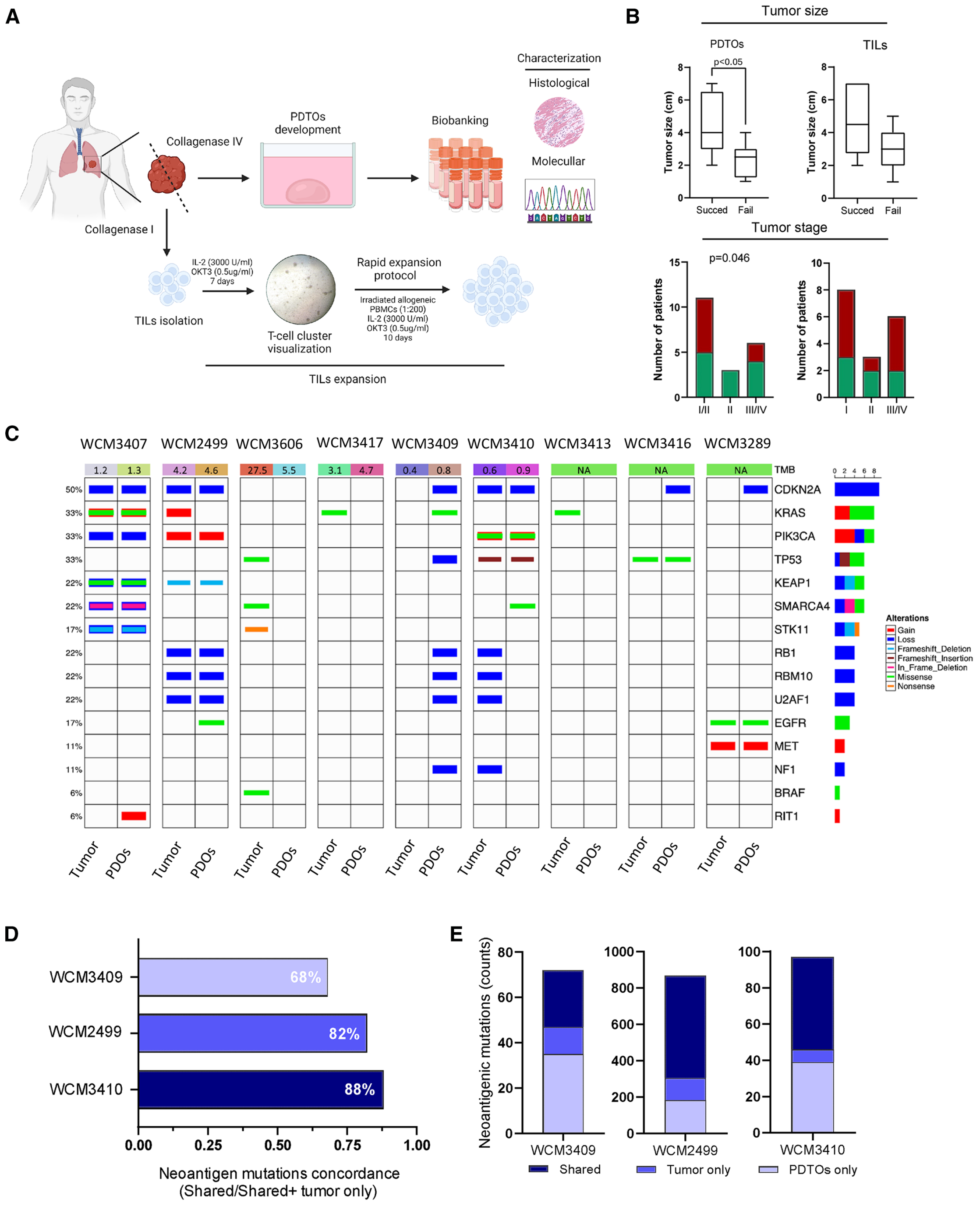

Figure 1. PDTO establishment, TIL isolation success rate, and PDTO characterization

(A) Tumor processing protocol for PDTO establishment and TIL isolation. NSCLC tumor resections ≥2 cm were split for parallel processing. For PDTOs, tumors were digested with collagenase IV, seeded in Matrigel domes, expanded, and biobanked. Histopathological review and molecular characterization confirmed concordance with the tumor of origin. For TILs, fragments were digested with collagenase I, plated in T cell media with IL-2 (3,000 UI/ml) and α-CD3 (0.5 μg/ml), and expanded via rapid expansion protocol (REP) when clusters appeared (~7 days). PBMCs were isolated from peripheral blood collected during resection.

(B) Associations between tumor size or disease stage and success rates. Tumor size differences between success and failure groups were assessed by Mann-Whitney test (boxplots show median and quartiles). Stage associations were evaluated by Chi-square test, with successful (green) and failed (red) case counts indicated.

(C) Oncoprint showing genomic concordance in driver genes between PDTOs and tumors, including mutations and copy number alterations in 19 NSCLC-relevant genes.

(legend continued on next page)

A scalable approach for assessing T cells effector functions in co-culture with PDTOs

Co-culture of PBMCs with autologous NSCLC- PDTOs has been shown to lead to an increase in the frequency of tumor-reactive IFN-γ+ T cells. To assess TIL anti-tumor reactivity and compare them with autologous PBMCs, we followed the same protocol with minor modifications.[26] Briefly, each T cell sample was divided into two groups, both cultured for 14 days in the presence of IL-2, but only one group had PDTOs added on days 0 and 7 (co-cultured group, CC). At day 14, T cells from both groups were harvested, re-plated, and co-cultured with PDTOs to perform functional assays to evaluate T cell tumor-killing capacity and IFN-γ secretion. Additionally, we added different ICIs (α-PD-1, α-PDL1, α-PD-1/PDL1, and α-TIM3) at days 0 and 7 of culture.

Dijskstra et al. reported that the frequency of IFN-γ + T cells after co-culture increased for most cases, yet the overall percentage of these cells was modest. This could potentially be related to the low sensitivity of intracellular cytokine staining (ICS) as the experimental readout for detecting tumor reactive T cells. Thus, in addition to ICS, we evaluated IFN-γ release using fluoroSpot (FS), since this technique is reported to be at least 500 times more sensitive than flow cytometry, enabling detection of antigen-specific T cells present at low clonal frequencies.[27] Overall, FS yielded better resolution (higher fold changes between basal and treated conditions) and higher sensitivity than ICS for evaluating anti-tumor reactivity. This higher sensitivity shown by FS translates into a significantly lower number of cells required compared to ICS, allowing an increase in the number of experimental conditions that can be assessed for a particular patient sample, including multiple drug combinations. Results of the ICS vs. FS comparison, including a cross-assay correlation analysis for validation, are displayed in Figure S2.

By analyzing IFN-γ secretion patterns, we were able to capture significant inter-patient heterogeneity in responses to different ICIs. Specifically, the addition of α-PD-1 and α-TIM3 triggered responses in most cases, with PBMCs showing a response of higher magnitude in 3 of 5 patients (Figure 2A).

In addition to IFN-γ secretion, we evaluated the cytotoxic activity of T cells after co-culture by the optimization of an image-based killing assay. After 12 h of culture, the percentage of apoptotic PDTOs in each experimental condition was heterogeneous among the different patients (Figures 2B and 2C). All patients responded to α-PD-1, which increasing killing, followed by α-TIM3, which modulated T cell responses in 3/5 cases. Of note, PBMCs exhibit higher cytotoxic activity than TILs after co-culture, except for WCM2499 (Figure 2C). By optimizing these downstream co-culture functional assays, we were able to systematically evaluate T cell responses under treatment with different ICIs, capturing inter-patient heterogeneity and highlighting the value of our experimental platform for immunotherapy screens.

To evaluate the impact of co-culture on TIL and PBMC functionality, we compared the cytotoxic activity of co-cultured T cells with those cultured for 14 days alone. We found that, when co-cultured, PBMCs displayed higher killing levels in the presence of α-PD-1 and α-TIM3 compared to the basal condition. In contrast, TILs showed lower basal killing levels after co-culture that were partially reversed with the addition of the different ICIs (Figure 2D). Consistently, expression levels of inhibitory receptors after co-culture were higher for both T cell sources, with TILs showing higher baseline and final expression levels (Figure S3A). These results suggest that the 14 day co-culture strategy might not be appropriate when using TILs as the source of T cells.

To confirm the detrimental effect of the 14 day co-culture on TIL cytotoxic activity and based on the premise that TILs constitute a T cell population already enriched in anti-tumor clones, as opposed to their PBMC counterparts, we compared TIL cytotoxic activity after the rapid expansion protocol (*ex vivo*, EV) with that recorded after 14 days of culture only with IL-2 (referred as cultured, C) or co-cultured with PDTOs (CC). We conducted these experiments only with WCM2499, WCM3606, and WCM3417 due to sample availability. Without anti-PD-1 addition, the basal killing capacity of TILs EV remained as low as in the other two culture conditions. However, when α-PD-1 was added, TIL tumor-killing capacity increased for all conditions, being significantly higher for TILs EV. Of note, even when the killing levels obtained by PBMCs after co-culture were similar to TILs EV and C after 12 h, 3 h after re-challenge with PDTOs, the killing capacity of TILs EV was significantly higher (Figure 2E). Overall, these results indicate that assessing TIL cytotoxicity EV improves T cell cytotoxic activity compared to 14-day cultures, confirming that no further culture is required to achieve measurable effector functions.

ICIs combinations screening: α-TIM3 and α-TIGIT enhance the effect of α-PD-1 on TIL effector functions

The upregulation of immune checkpoints on T cells after co-culture, as well as the heterogeneous expression of their ligands on matching PDTOs (Figure S3B), make the co-culture systems potentially valuable tools for evaluating combination immunotherapies. As a proof of concept, we assessed WCM2499, since the PDTO expressed most of the inhibitory ligands and the TILs displayed significant effector functions.

In this assay, we combined different concentrations of α-PD-1 with α-TIM3, α-TIGIT, or α-LAG3. In agreement with the experiments shown in Figure 2A, increasing concentrations of α-PD1 boosted IFN-γ release. α-TIM3 increased IFN-γ secretion by itself at 10 and 20 μg/ml and enhanced the effect of α-PD-1 at both concentrations. α-TIGIT addition only boosted IFN-γ release at 20 μg/ml when α-PD-1 was added (at 20 μg/ml). α-LAG3 addition did not have any effect on IFN-γ production (Figure 3A , left). The tumor-killing assay reflected similar trends

(D) Neoantigen-associated mutations predicted from WES data for three cases. Concordance was calculated as shared mutations/(shared + tumor-exclusive mutations).
(E) Counts of neoantigenic mutations present in tumor only, PDTOs only, or shared.
See also Figure S1 and Tables S1–S3.

Table 1. Tumor of origin histopathological features and success of PDTO establishment and TIL expansion

Patient ID	Original histology	WHO 2021 classification	Patterns	PDTOs establishment(YES/NO)	TILs expansion(YES/NO)	Tumorp urity(PDTO/tumor)
WCM3602	LUAD	invasive non-mucinous	solid, acinar, lepidic	NO	NO	N/A
WCM3603	LUAD	invasive non-mucinous	solid	NO	YES	N/A
WCM3604	LUAD	invasive mucinous	mucinous dominant and micropapillary	NO	NO	N/A
WCM3605	LUAD	invasive non-mucinous	acinar, micropapillary, and lepidic	NO	NO	N/A
WCM3607	LUAD	invasive non-mucinous	acinar and lepidic	NO	NO	N/A
WCM3608	LUAD	invasive mixed mucinous and non-mucinous	acinar, solid, micropapillary, lepidic and mucinous	NO	NO	N/A
WCM3083	LUAD	invasive non-mucinous	acinar	NO	NO	N/A
WCM3407	LUAD	invasive non-mucinous	micropapillary and papillary	YES	NO	99%/50%
WCM2499	LCNEC	LCNEC	LCNEC	YES	YES	85%/71%
WCM3606	LUAD	invasive non-mucinous	micropapillary and acinar pattern	YES*	YES	N/A
WCM3417	LUAD	invasive mucinous	mucinous	YES*	YES	N/A
WCM3409	LUAD	mixed mucinous and non-mucinous invasive	micropapillary, solid, acinar, mucinous and papillary	YES	YES	90%/N/A
WCM3410	LUAD	mixed mucinous and non-mucinous invasive	micropapillary, acinar, mucinous and papillary	YES	YES	99%/21%
WCM3413	LUAD	invasive non-mucinous	micropapillary, acinar and lepidic	YES	NO	50%/35%
WCM3066	LUAD	invasive non-mucinous	solid and micropapillary	NO	NO	N/A
WCM3416	LUSC	invasive SqCC	invasive SqCC	YES	YES	50%/50%
WCM3289	LUAD	invasive non-mucinous	solid	YES	NO	50%/30%

* Indicates samples that did not reach the “growing well” status, but for which enough material was obtained to perform co-culture assays. Percentages or tumor purity were estimated by WES and Clonet. N/A indicates not available. LUAD, adenocarcinoma; LCNEC, large neuroendocrine carcinoma; LUSC, squamous cell carcinoma.

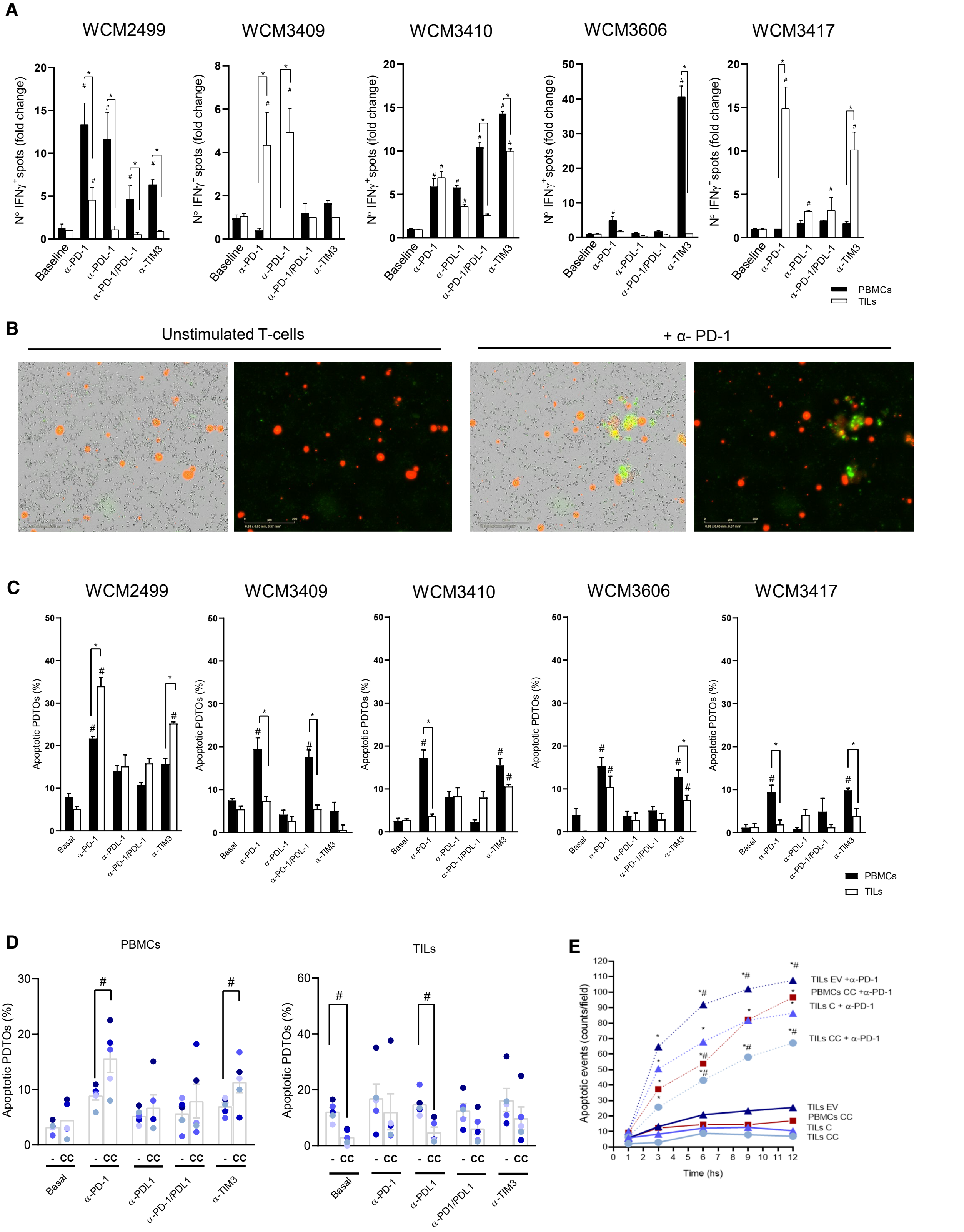

(legend on next page)

as IFN-γ secretion. The highest concentration of α-TIM3 enhanced T cell cytotoxic activity in the presence of α-PD-1. With α-TIGIT, T cell-mediated tumor killing significantly increased only in combination with α-PD-1 at 20 μg/ml. α-LAG3 addition did not modulate cytotoxic activity (Figure 3A, right). These results highlight the potential of the PDTOs/TIL co-culture to address combinations of ICIs in a patient specific fashion.

Sequential combination therapy screening: Pre-treatment with PI3K inhibitors sensitized KRAS (G12A) mutant NSCLC-PDTOs to immunotherapy

Targeted therapies have become a research focal point due to their specificity to tumor cells and minimal adverse effects in comparison to chemotherapies.To establish the utility of our PDTO/TIL co-culture system for screening combinations of drugs, including immune therapies, we performed a drug screening employing two PDTO lines bearing KRAS G12A mutations, a low-frequency KRAS mutation for which there are no clear therapeutic options. Additionally, each of these cases belong to a particular subset of KRAS mutants based on the co-occurrence of alterations in TP53 (WCM3409) or STK11 (WCM3407).[28] We evaluated whether pre-treatment with different targeted inhibitors could sensitize PDTOs to T cell-mediated killing in the presence of ICIs.

First, we evaluated PDTO sensitivity to 23 different targeted inhibitors against EGFR (lapatinib, osimertinib, dacomitinib, afatinib, erlotinib, and gefitinib), PI3K (idelalisib, copanlisib, PI-103, buparlisib, GSK2636771, parsaclisib, and erganelisib), mTOR (temsirolimus, rapamycin, and everolimus), ERK (ulixertinib), Raf (AZ628 and dabrafenib), Ras (AMF510), and MEK (binimetinib, trametinib, and selumetinib). WCM3409 was sensitive to 7 inhibitors: 4 EGFR inhibitors (osimertinib, afatinib, dacomitinib, and lapatinib), 2 PI3K inhibitors (buparlisib and copanlisib), and 1 MEK inhibitor (trametinib). WCM3407 was sensitive to 9 inhibitors: 5 EGFR inhibitors (lapatinib, osimertinib, erlotinib, afatinib, dacomitinib, and gefitinib) and 3 PI3K inhibitors (copanlisib, PI-103, and buparlisib). Dose-response curves for the hit compounds are shown in Figure S4A.

To address whether these targeted therapies could enhance the effect of α-PD-1 and α-TIM3, we tested sublethal drug concentrations (EC10, EC20, and EC25) calculated from the full dose-response curves (Figures S4B and S4C). A schematic experimental timeline is depicted in Figure 3B. Fold increases in T cell killing capacity over the condition without targeted agents are displayed in Figure 3C for WCM3409 and WCM3407. In cases where the fold increase was ≥2, we analyzed differences in the percentages of apoptotic PDTOs to determine whether the recorded increases were statistically significant. We found that the addition of the PI3K inhibitors, copanlisib and buparlisib, increased killing levels in the presence of α-PD-1 and α-PD-1 plus α-TIM3 (5 μg/ml) for WCM3409 (Figure S4D). The increase in the percentage of apoptotic PDTOs was significantly pronounced for buparlisib and copanlisib. In the case of WCM3407, buparlisib, copanlisib, and PI-103 significantly enhanced the killing percentage when α-PD1 was added alone or in combination with a high concentration of α-TIM3 (Figure S4D).

Since the concentrations of the targeted therapies used in these experiments were below EC25, the potentiation of the effect of the different ICIs on PDTO killing should not be directly related to the exposure of antigens as a consequence of tumor cell death. Therefore, we performed a cytokine array on the supernatant of PDTOs treated with the different PI3K inhibitors to determine whether there were variations in the immunoregulatory molecules released by the tumor cells. In both PDTOs, we observed that the addition of the PI3K inhibitors reduced the secretion of several cytokines capable of modulating T cell activity, including IL- 8 and macrophage migration inhibitory factor (MIF) in both cases, IL-6, IL-13, IL-18, and PAI-1 in WCM3409, and CXCL1 in WCM3407 (Figure 4E). These results highlight another advantage of co-culture models, in particular our sequential combination therapy screening, which is the identification of potential microenvironmental signals that could be impairing ICI efficiency, leading to the identification of potential new therapeutic strategies.

Reprogramming healthy donor macrophages into PDTO-specific TAMs

Chemotherapy resistance in NSCLC is influenced by several factors, with TAMs playing a critical role in this process.[29] To explore this, we focused on optimizing strategies to reconstitute

Figure 2. Optimization of functional assays for evaluating T cell effector functions and modulation by immune checkpoint inhibitors after co-culture with PDTOs

(A) IFN-γ secretion by FluoroSpot. Bar graphs show mean fold change ±SD of IFN-γ-producing T cells ($n = 3$) at baseline and with different monoclonal antibodies (mAbs). Frequencies are displayed as fold change of $CD8^+$ IFNγ+ spots relative to the no-ICI condition. PBMCs (black bars) and TILs (white bars) are shown ($n = 5$). (*) Kruskal-Wallis test with Dunn's post-test ($p < 0.05$).

(B) Schematic of the image-based tumor killing assay. Far red-stained PDTOs ($3–5 \times 10^4$) were seeded in 96-well plates. T cells were added (3:1 E:T ratio) in media containing NucView488 Caspase-3 substrate (5 μM) and co-cultured 12 h with hourly imaging. Apoptotic PDTOs (yellow events) were quantified using Incucyte S3 (3 fields/well). Scale bars, 200 μm.

(C) Apoptotic PDTOs (%) after 12h co-culture for PBMCs (black) and TILs (white) by patient. Data are shown as mean ± SEM of 3 fields/condition. Kruskal-Wallis test with Dunn's post-test ($p < 0.05$).

(D) Tumor killing by T cells cultured for 14 days with IL-2 alone (−) or IL-2+PDTOs (co-cultured; CC). Apoptotic PDTOs (%) after 12 h is shown for PBMCs (left) and TILs (right) ± ICIs. # indicates significant difference (Mann-Whitney test, $p < 0.05$).

(E) TIL killing under different culture conditions: co-cultured 14 days (CC, light blue), IL-2 alone 14 days (C, purple), or post-REP (*ex vivo*/EV, dark blue). PBMC average is shown (red). Mean apoptotic events (counts/field) for WCM2499, WCM3417, and WCM3606 TILs at baseline (solid) or with α-PD-1 (20 μg/ml, dotted) at 1, 3, 6, 9, and 12 h. Kruskal-Wallis with Dunn's post-test ($p < 0.05$).

* indicates difference from respective no-ICI; # indicates difference from TILs C+α-PD-1.

See also Figure S2.

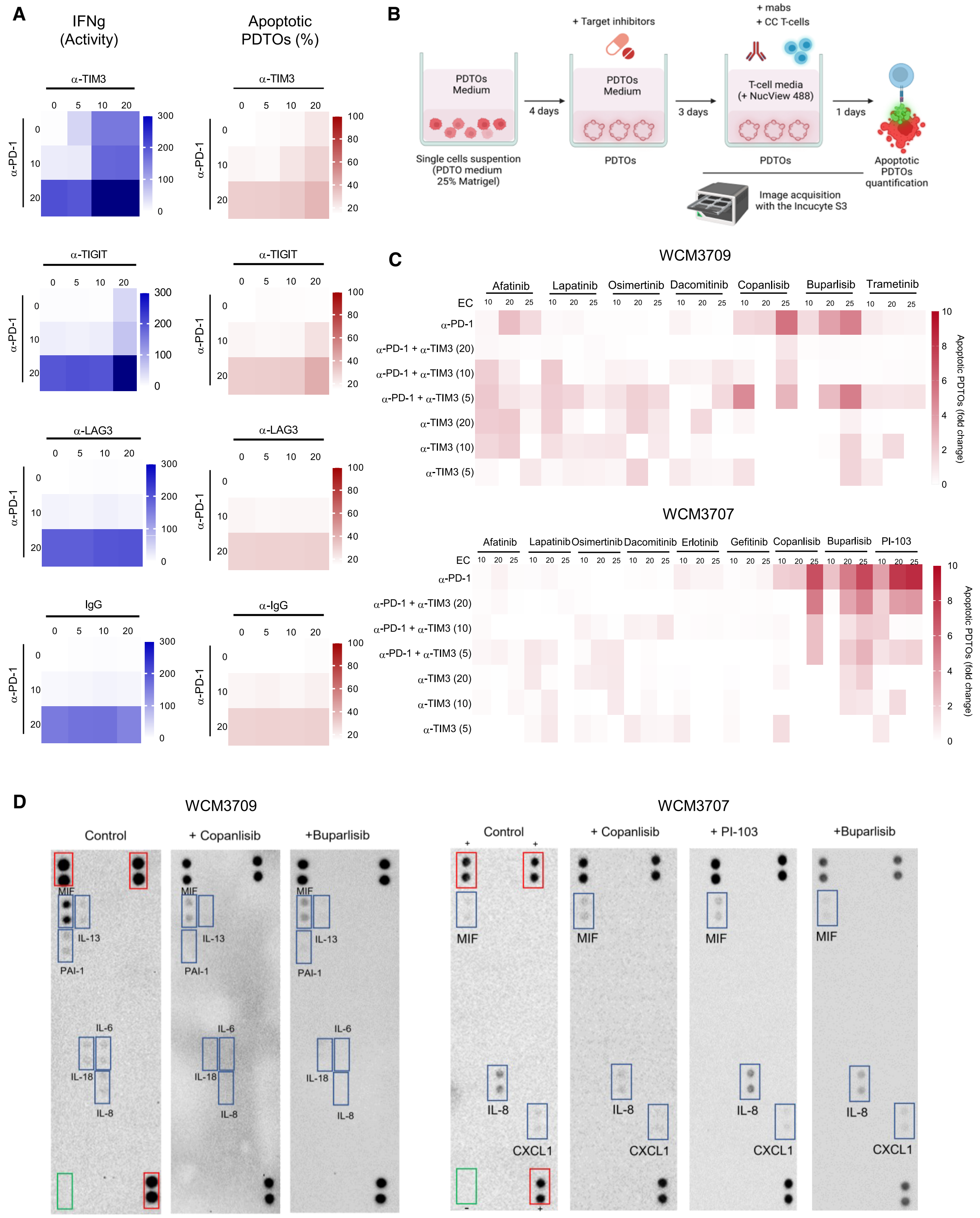

Figure 3. Assessing therapeutic combinations in PDTO-T cell co-cultures

(A) WCM2499 TILs EV challenged with PDTOs (1:5) in the presence of α-PD-1 (10 and 20 μg/ml) combined with α-TIM3, α-TIGIT, or α-LAG3 (5, 10, 20 μg/ml). Heatmaps show IFN-γ activity (left) and T cell-mediated PDTOs killing (% apoptotic, right).

(legend continued on next page)

these key components of the TIME within our PDTO cultures. Given the challenge of culturing monocytes at large scale, we isolated monocytes from healthy donor leukopaks, differentiated them into M0 macrophages, and then polarized them into TAMs by co-culturing with PDTOs for 48 h. Additionally, we differentiated and polarized macrophages into distinct canonical subsets (M1, M2a, M2c, M2d) *in vitro*, which allowed us to compare their transcriptional profiles with those of TAMs polarized with PDTOs (Figure S5A). We analyzed the expression of genes associated with M2 (CD206, CD163, MerTK, IL-10, TGFβ), M1 (CD80, HLA-DR, CD64, IL1β, TNF-α), and lung-specific macrophage markers (PLAU, MARCO, TREM1, CHI3L1, CHI3L2, SPP1, and APOE) (Figure 4A). Notably, the gene expression profiles of each PDTO-derived TAM differed but shared similarities with the M2c and M2d canonical subsets. When examining the overall transcriptional profile of the *in vitro* polarized TAMs, we observed that each subset had its own distinct set of differentially expressed genes, with only 76 genes being common across all subsets (Figure 4B).

To identify additional markers for NSCLC TAM subsets, we focused on the shared genes encoding transmembrane receptors and assessed their expression by flow cytometry. Polarized macrophages were stained with a panel of surface markers, including M1 (CD80, HLA-DR), M2 (CD163, CD206), lung macrophages (TREM1, MARCO), and three upregulated transmembrane receptors (CD24, CD66e, and CD66c) identified in the RNA-seq data for all PDTO-polarized TAMs, for which we were able to obtain positive antibody staining. Dimensionality reduction analysis revealed the diversity of macrophage populations across the different canonical subsets, as well as in the TAMs (Figure 4C). Using the Phenograph algorithm, 25 macrophage clusters were identified, with cluster 13 being notably overrepresented in the PDTO-polarized TAM samples (Figure 4D). This cluster was primarily characterized by high expression of CD163 and TREM1 (Figure 4E), with TREM1 having been previously identified as a marker of NSCLC TAMs and associated with poorer clinical outcomes.

Different TAM and PDTO co-culture systems enabled the dissection of soluble and cell-cell signals effects: TAMs addition to culture boosted PDTO growth

To explore the effects of soluble and cell-cell signaling on PDTO growth, we utilized different TAM and PDTO co-culture systems. We investigated how TAM addition influences PDTO growth. When co-culturing PDTOs with immune cells, it is crucial to assess the extracellular matrix (ECM) composition to ensure optimal PDTO growth and immune-cell motility. In our initial approach, we tested conventional PDTO growth conditions using 66% Matrigel domes and observed that macrophages were unable to penetrate the Matrigel (Figure S5B). Given the importance of soluble factors in the tumor-promoting properties of TAMs, we next assessed PDTO growth over a 12-day period, with and without the addition of TAMs. PDTO cells were plated in 66% Matrigel domes and cultured for 3 days. On day 3, TAMs were added at a 1:3 ratio (PDTO cells:TAM) and cultured for an additional 12 days. Daily images were captured using an Incucyte S3, and the total PDTO area was recorded as a measure of growth (Figure 5A). We observed that when TAMs were present, PDTOs exhibited enhanced growth, both in terms of rate and total area (Figures 5B and 5C).

To enable cell-cell interactions between PDTOs and TAMs, we tested different Matrigel concentrations ranging between 20% and 50%. We found that concentrations between 20% and 30% allowed optimal motility, enabling TAMs to move freely. However, at higher concentrations, TAMs were unable to penetrate the domes (Figure S5B). Based on these findings, we chose to proceed with the 20% Matrigel condition. To facilitate the identification of different cell types, PDTOs and TAMs were stained with distinct CellTrace dyes prior to co-culture. CellTrace Blue was used to stain PDTO cells, which were seeded in a 20% Matrigel layer. CellTrace Far Red was used to stain TAMs, which were added 3 days later and cultured for an additional 12 days. On day 12, the plates were imaged, and the fold change in the total blue area was evaluated as a measure of PDTO growth (Figure 5C). Consistent with our previous observations, the addition of TAMs resulted in a higher fold change in the total blue area, indicating enhanced PDTO growth (Figure 5D). Co-culture systems demonstrated that TAMs promote PDTO growth through a combination of soluble signals and direct cell-cell contact, with optimal interaction achieved by tuning the ECM composition.

Co-culture systems capture TAM-mediated resistance to gemcitabine

In a proof-of-concept experiment, we evaluated whether the addition of TAMs to PDTO cultures modified their sensitivity to chemotherapy. Tumor cells were plated in 20% Matrigel and incubated for 3 days. On day 3, TAMs (1:3, PDTO cells:TAM) and drugs were added and incubated for 5 days. On day 5, green fluorescence was quantified as a measure of cell death. Of the three agents we assessed, TAMs only modulated PDTO sensitivity to gemcitabine, reducing killing at lower concentrations of the drug. No effect was observed on paclitaxel sensitivity, and PDTOs were not sensitive to carboplatin (Figure 6A).

TAMs' effect on PDTO sensitivity to gemcitabine was not uniform. The effect was more pronounced in WCM2499 PDTOs, where a shift in the dose-response curve was observed at low concentrations. For this reason, we selected WCM2499 to test whether our imaging strategy, described in Figure 5D, could be adapted to recapitulate gemcitabine resistance results obtained

(B) Tumor killing assay for combined targeted therapy/ICI screening. Far-red-stained tumor cells plated in 25% Matrigel for 4 days to form PDTOs. Targeted inhibitors were added at day 4 and incubated for 3 days. mAbs and T cells were added in media with NucView488 at day 7 and imaged overnight using Incucyte S3.

(C) Fold increase in tumor killing with targeted agents (EC10, EC20, EC25) calculated relative to the ICIs-only condition.

(D) Cytokine array of PDTO culture supernatants treated for 48 h with PI3K inhibitors (copanlisib, buparlisib, and PI-103) at EC25. Red: positive controls; green: negative control; blue: detected cytokines.

See also Figures S3 and S4.

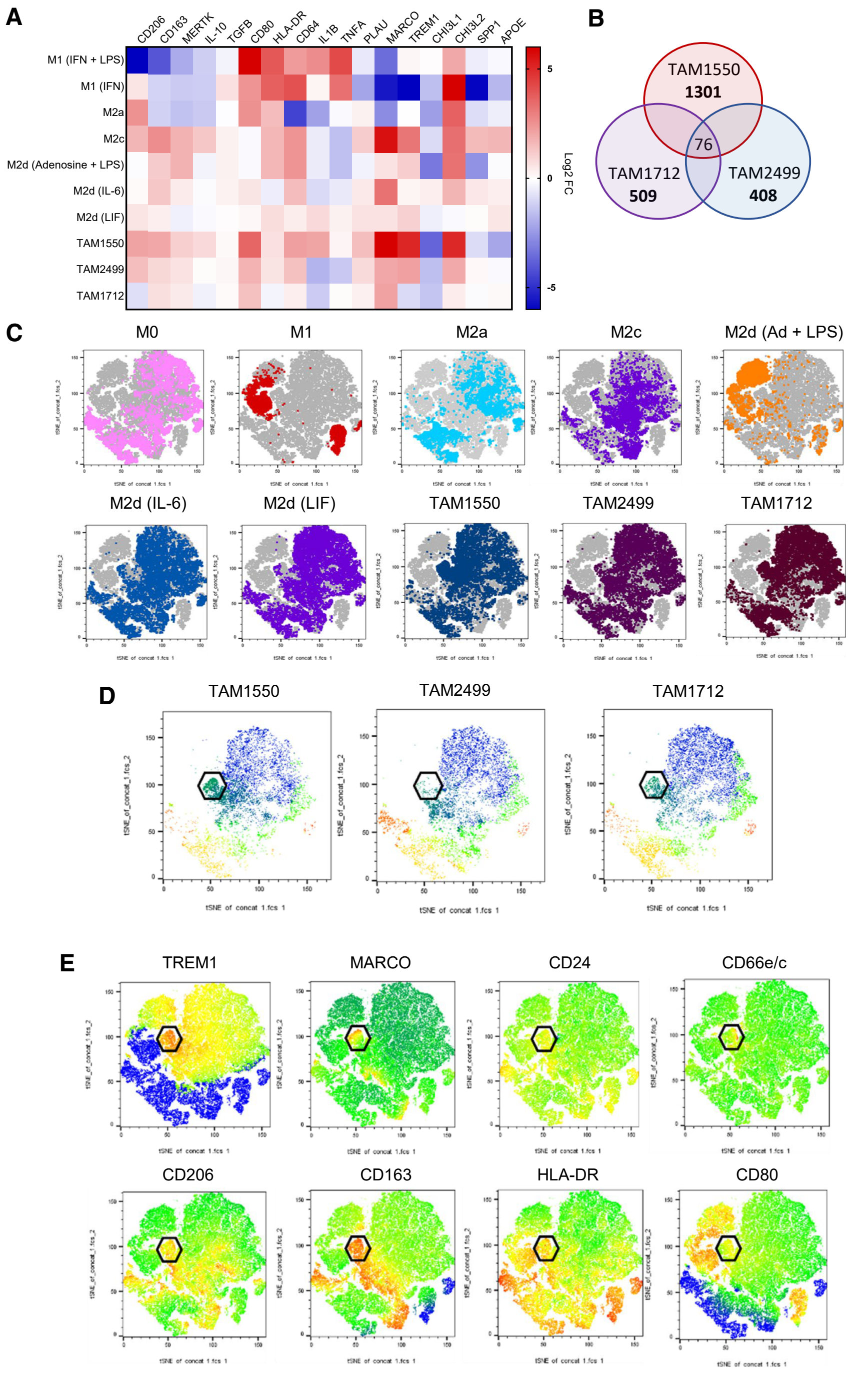

Figure 4. *In vitro* polarization of macrophages into PDTO-specific TAMs

(A) Gene expression of M1/M2 conventional markers and lung-associated macrophage markers in polarized macrophages. Expression levels were normalized to the M0 baseline and are shown as Log2 fold change.

(B) Venn diagrams showing differentially expressed genes in each TAM sample and those shared across all samples (76 genes).

(C) Dimensionality reduction showing macrophage population diversity. Canonical subsets and PDTO-induced TAMs are colored by subset.

(D) Dimensionality reduction and clustering of PDTO-polarized samples. Phenograph identified 25 clusters; cluster 13 (hexagon) is significantly enriched in PDTO-polarized TAMs.

(E) Marker expression heatmaps across clusters showing M1/M2 markers, lung-associated macrophage markers, and differentially expressed transmembrane proteins on TAMs. Color scale ranges from blue (no expression) to orange (high expression), based on mean fluorescence intensity.

See also Figure S5.

by fluorometry. CellTrace Blue tumor cells were plated in 25% Matrigel and incubated for 3 days. On day 3, CellTrace Far Red TAMs (1:3, PDTO cells:TAM) and gemcitabine were added and cultured for 5 additional days. On day 5, NucGreen, a DNA-binding agent that only penetrates cells when plasma membrane integrity is compromised, was added 30 min before imaging. Average green fluorescence intensity per field was recorded as a measurement of cell death (Figure 6B). Our results showed that TAM addition significantly reduced PDTO death at 0.128 and 0.64 nM of gemcitabine. For the higher concentration assessed, there is a trend for reduction, but it is not statistically significant (Figure 6B). Our image-based results not only recapitulate the results obtained by the fluorometric assay but also confirm that most of the drug-mediated killing happened in PDTOs.

Here, we developed a strategy for the *in vitro* differentiation of patient-/tissue-specific TAM populations and showed that their reconstitution in PDTO culture promotes tumor cell growth and modifies sensitivity to chemotherapy. Our results delineated, for the first time, a strategy for TAM reconstitution in PDTO culture suitable for high-throughput drug screens.

DISCUSSION

PDTOs have created a valuable platform for testing therapeutic agents and hold great promise for personalized medicine. PDTOs have already been evaluated in the context of high-throughput drug screening and modeling chemo- and targeted therapies.[17,20,30–32] Nevertheless, there has been no report to date proposing TIME reconstitution approaches for high-throughput drug screens.

Our initial attempts to process entire tumor samples with a single collagenase type revealed complementary limitations: collagenase I enabled TIL isolation but prevented PDTO establishment, while collagenase IV yielded viable PDTOs but failed to

CellPress
OPEN ACCESS

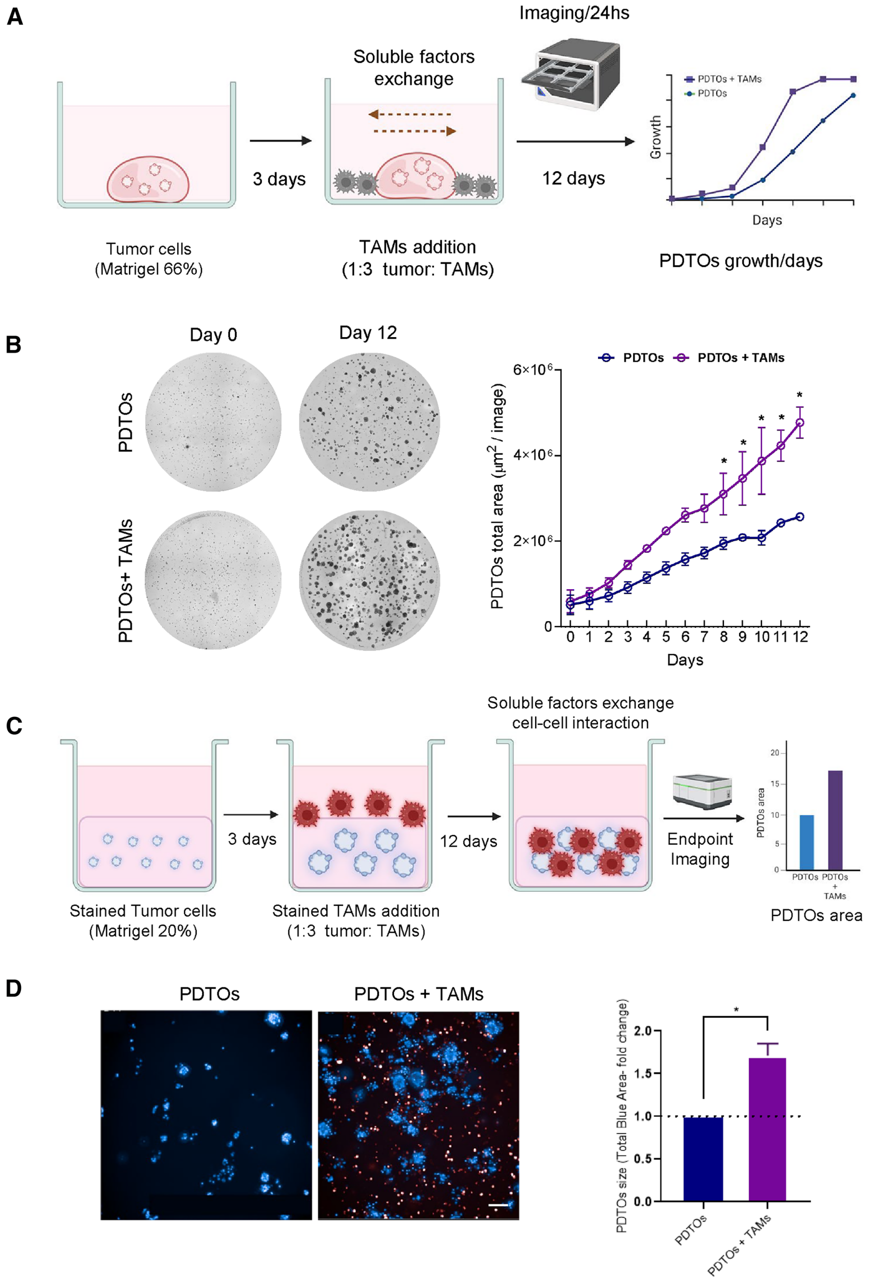

Figure 5. Impact of TAM reconstitution on NSCLC-PDTO growth

(A) Effect of TAM-derived soluble factors on PDTO growth. PDTOs were seeded in 66% Matrigel droplets; TAMs were added outside the droplets (1:3 ratio) after 3 days and co-cultured for 12 days, with daily area recording (Incucyte S3).

(B) Representative images of PDTOs ± TAMs at days 0 and 12 (left). Total PDTO area curves for PDTOs+TAMs (violet) and PDTOs alone (blue) are shown as mean ± SEM ($n = 3$). Kruskal-Wallis test with Dunn's post-test ($p < 0.05$). * different from no-TAM condition (right).

(C) Direct TAM-PDTO co-culture schematic. PDTOs were seeded in 20% Matrigel; TAMs (CellTrace Far Red) were added (1:3 ratio) after 3 days. At day 12, wells were imaged, and the total blue area (PDTOs) were quantified.

(D) Representative images of PDTOs (blue) ± TAMs (red) at day 12 (left). Total PDTO area quantification is shown as mean fold change relative to the no-TAM condition (mean ± SEM, $n = 3$). Kruskal-Wallis ($p < 0.05$). * indicates significant difference (right). Bar represents 50 µm.

See also Figure S5.

isolate TILs. Collagenase I's higher proteolytic activity likely facilitates more efficient digestion of the dense, fibrotic stroma in lung tumors, enabling better lymphocyte release but compromising the epithelial cell viability required for PDTO culture. Conversely, collagenase IV's gentler digestion preserves the cell viability necessary for PDTO establishment but appears insufficient to access embedded lymphocyte populations. This prompted our hybrid approach of splitting tumor samples to leverage the optimal enzyme for each application.

A key question in designing experiments for PDTO and T cell co-culture is the choice of T cell source: PBMCs or TILs. PBMCs can be easily isolated from peripheral blood samples, and even when the clonality of tumor-specific T cells is low, it can be increased by co-culture with PDTOs[26,33] The clones expanded with this method are shared, to some extent, with those present in TILs, as has been described for PBMCs co-cultured with pancreatic PDTOs[33] On the other hand, TILs are employed in cell therapy for various solid tumors because they are rich in tumor-reactive T cells.[34,35] Their value as a predictive biomarker of immune checkpoint blockade response is being evaluated in several types of solid tumors, including NSCLC.[7,36] Here, we compared autologous TIL and PBMC effector functions after co-culture and found that long co-culture with PDTOs is detrimental for TILs.

During the process of TIL isolation and expansion, T cells undergo several rounds of amplification in response to polyclonal stimuli such as IL-2 and α-CD-3. Furthermore, as part of the traditional procedures for TIL manufacturing, they are subjected to a selection phase in which T cells are assessed for specific tumor recognition—in our case, the 14 day co-culture with autologous PDTOs. This whole process makes TILs prone to exhaustion, limiting their functionality.[37,38] Alternative approaches are being evaluated to improve TIL obtention, including protocols such as the replacement of IL-2 with IL-15 and IL-21, and avoiding selection steps *in vitro* by directly employing post -REP TILs, called young TILs.[35,39] Following this approach, we found that TILs post-REP (EV) display higher cytotoxic activity than their after-co-cultured counterparts. Since the

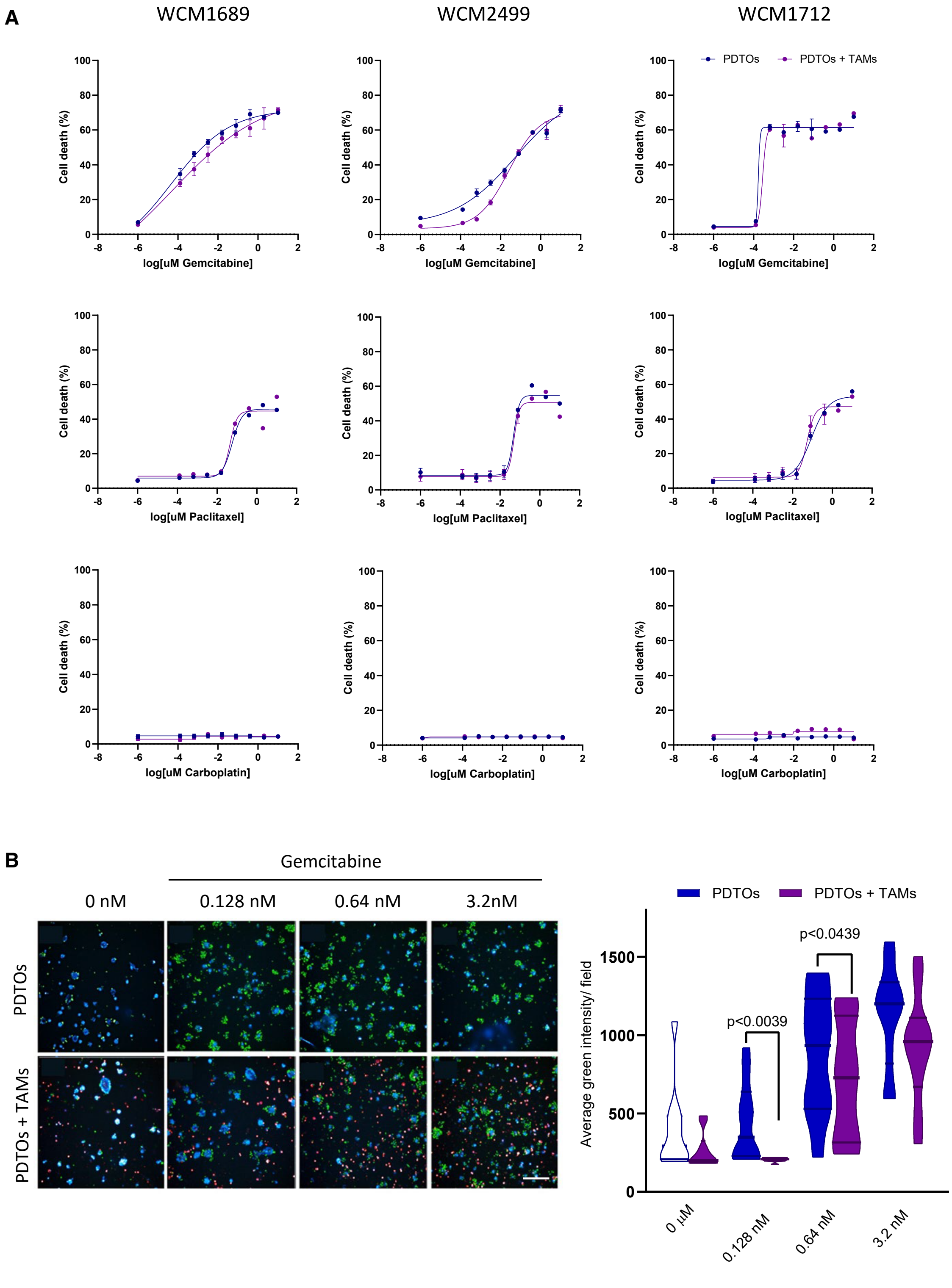

Figure 6. Effect of TAM addition on PDTO chemotherapy sensitivity

(A) Drug response curves of PDTOs ± TAMs for gemcitabine (upper), paclitaxel (middle), and carboplatin (lower). PDTOs were seeded in 66% Matrigel ± TAMs (1:3). Drugs were added at day 3 and incubated for 3 days. CellTox Green staining was performed at day 6 per the manufacturer's instructions. Cell death (%) was calculated using 10 μM staurosporine-treated PDTOs as 100% death. Curves show mean ± SEM (*n* = 2).

(legend continued on next page)

cytotoxic levels recorded after 12 h by TILs EV and PBMCs after co-culture were similar, the decision of which source to use for the assays will depend on the tumor type and the type of sample, biopsy or resection. We encourage the use of TILs, if possible, because the translational implications of the screening would be more valuable, and the time frame required to obtain data would be significantly shorter. Nevertheless, given the low success rate of their obtention, expanding tumor-reactive T cells from PBMCs remains a reasonable alternative.

Currently, PD-1 blockade is approved for use in first- and second-line treatment of advanced non-squamous NSCLC. Unfortunately, many patients with NSCLC do not respond or do so only briefly and then relapse. The mechanisms behind this are being extensively investigated. One major known mechanism is the expression of other inhibitory receptors on TILs after the initial upregulation of α-PD-1.[39,40] As an increasing number of checkpoint molecules are discovered on exhausted T cells, it is essential to understand which of them has a dominant effect in order to design effective combination strategies that can become increasingly complex with significant toxicity profiles. To achieve this goal, robust human-based pre-clinical models are needed. The expression of different exhaustion molecules on TILs post-REP and on PBMCs after co-culture offers an opportunity for the evaluation of ICI combinations.[41,42] Among the exhaustion markers evaluated in our patient cohort, TIM3 and TIGIT were the receptors with the highest expression levels. Previously, the combinations of α-PD-1 blockade with α-TIM3 or α-TIGIT were assessed in a restricted human TIL adoptive transfer model employing an engineered lung cancer cell line (A549) expressing NY-ESO and HLA-A2 (A549-A2-ESO) and the Ly95 T cell expressing the NY-ESO-1 TCR. In that model, the authors reported that the combination of α-PD-1 either with α-TIM3 or α-TIGIT reinvigorates Ly95 T cell reactivity and tumor control.[43] Using our co-culture platform, we were able to achieve similar results employing WCM2499 TILs EV and autologous PDTOs. The combination of α-PD-1 blockade with α-TIM3 and α-TIGIT increased PDTO killing and IFN-γ release. Of note, here we only explored well-known immune-checkpoint receptors but combining these models with next-generation sequencing strategies and molecular editing tools would enable the identification of new molecules that could be targeted to potentiate current therapies or develop new ones.

The identification of subsets of patients with oncogenic drivers has transformed the treatment of NSCLC, particularly for those patients whose tumors harbor mutations in EGFR or fusions involving ALK, RET, and ROS1 kinases.[12,44] However, these genomic alterations occur in a relatively small percentage of patients with NSCLC, mainly LUAD, and when actionable, the efficacy of the available targeted drugs is limited due to the development of acquired resistance through different molecular mechanisms.[9] Therapeutic approaches combining target inhibitors and immunotherapies are being evaluated for these patient subsets, constituting a promising therapeutic field.[7] Nevertheless, the development of therapeutic strategies for *KRAS* mutants, the most common oncogenic driver in NSCLC, has not been successful so far.[28] The development of effective therapies for *KRAS* -mutant NSCLC is challenging due to heterogeneity in their biology and therapeutic responsiveness.[28]

Besides the alteration in *KRAS*, the co-occurrence of other genomic alterations in *TP53*, *STK11*, and *CDKN2A/B* defines particular patient clusters (named KP, KL, and KC, respectively).[28] One of the many molecular differences between the three clusters is the signature of immune-related genes. Gene set enrichment analysis (GSEA) and Ingenuity Pathway Analysis highlighted gene sets associated with activation of antitumor immunity and immune tolerance/escape as prominent modules of the KP cluster. In contrast, KL tumors demonstrated a comparative lack of immune system engagement, whereas KC tumors demonstrated a mixed picture.[28]

In a proof-of-concept experiment, we used WCM3409 and WCM3407, both LUAD lines carrying a low-frequency *KRAS* mutation (G12A) and belonging to the KP and KL clusters, respectively. In an initial screening phase, we evaluated their sensitivity to a set of 23 inhibitors targeting EGFR, PI3K, mTOR, ERK, Raf, Ras, and MEK. Both lines were mostly sensitive to EGFR and PI3K inhibition. PI3K inhibitors significantly enhanced killing mediated by α-PD-1 blockade and α-TIM3 in both cases.

It has been reported that PI3K inhibition increases immunotherapy effectiveness, either by acting directly on tumor cells, making them more sensitive to immune recognition, or by enhancing T cell effector functions.[45,46] In addition, *KRAS* mutations have been associated with tumor-promoting inflammation and with the secretion of immune-suppressive cytokines.[47,48] Considering this, we evaluated in our sequential co-culture system whether the enhancement of ICI effects on T cell-mediated tumor killing, when PDTOs were pre-treated with PI3K inhibitors, was related to modulation of the cytokine profile secreted by the PDTO cells. Analyzing the supernatant of PDTOs treated with PI3K inhibitors for 48 h, we observed that both patients shared a common alteration: a reduction in the amount of IL-8 released by tumor cells. IL-8 not only promotes NSCLC cells growth and survival but also interferes with T cell function by inducing upregulation of PDL-1 on tumor cells and inducing apoptosis in subsets of effector $CD8^+$ T cells.[49,50] In addition to IL-8, IL-6 and MIF were the only cytokines detected that could be contributing to impaired T cell functions in our system, since they are capable of directly affecting T cell effector function.[51,52]

Overall, therapy resistance remains a significant challenge in the treatment of NSCLC, driven not only by intrinsic genetic alterations within the tumor, but also by changes in the TIME. Besides T cells, TAMs also play a pivotal role in therapeutic failure. Their phenotype is a critical determinant of their impact on tumor progression, with the spectrum of TAM phenotypes extending well beyond the traditional M1 vs. M2 dichotomy.[53,54] The M2 phenotype itself includes several subtypes (M2a, M2b, M2c, and M2d), each associated with distinct biological processes, such as tissue repair, immune suppression, immunoregulation, and tumor

(B) Image-based gemcitabine sensitivity assay. Tumor cells were seeded in 20% Matrigel ± TAMs for 3 days. Gemcitabine was added at day 3 and incubated for 3 days. NucGreen was added at day 6 for 30 min before imaging (4 fields/well). Representative images of PDTOs (blue) ± TAMs (red) at day 6 across gemcitabine concentrations (0–3.2 nM) (left). PDTO cell death is shown as mean green intensity/field with Q1 and Q3 (right). Kruskal-Wallis test with Dunn's post-test ($p < 0.05$). * indicates significant difference. Scale bar indicates 50 μm.

progression.[54] The complexity of microenvironmental cues further enhances the phenotypic diversity of TAMs within specific tumor environments. In NSCLC, a subset of TAMs expressing TREM1+ has been identified, with the presence of this population correlating with tumor growth promotion and poorer clinical outcomes.[55] Our *in vitro* polarization strategy enables the generation of PDTO-specific TAMs, each characterized by distinct sets of differentially expressed genes. Despite these variations, all TAMs share phenotypic features with M2c and M2d subsets, along with the expression of a lung-specific TAM subset marked by TREM1+.

Several research groups have previously acknowledged the relevance of TAMs in tumor biology, particularly regarding treatment sensitivity, and have developed various co-culture strategies.[56–58] However, these approaches are not easily adaptable to high-throughput drug screening due to limitations such as the need for microfluidic devices, the use of patient-derived peripheral blood monocytes (which limits cell availability due to cell number), or reliance on complex experimental readouts. Our co-culture strategy allows for the scalability of assays to assess the impact of TAMs on chemotherapy efficacy. In our proof-of-concept experiment, we not only performed an in-depth characterization of the in vitro-generated PDTO-specific TAMs but also screened PDTOs for sensitivity to paclitaxel, carboplatin, and gemcitabine using two different experimental readouts, fluorometric and image-based. Notably, we identified one case where TAM reconstitution led to significantly different results, highlighting the potential influence of TAMs on patient-specific chemotherapy response.

While our study focuses on reconstituting individual TIME components (TILs and TAMs), we recognized that the native tumor microenvironment contains additional cellular populations, including tumor-associated neutrophils, dendritic cells, and cancer-associated fibroblasts, that contribute to therapy responses. Our single-component approach was designed to establish scalable, controlled systems suitable for dissecting specific TIME-mediated mechanisms of drug resistance. Future iterations should integrate multiple TIME components to more comprehensively model immune-tumor-stromal interactions. Additionally, prospective studies correlating PDTO-TAM co-culture predictions with clinical treatment responses could validate these models for clinical decision-making and identify patients more likely to benefit from TAM-targeting combination strategies.

In this manuscript, we present several strategies for reconstituting different components of the TIME for therapeutic modeling. We emphasize the development of reproducible and scalable co-culture systems, as these are crucial for ensuring that the models can effectively identify the most effective compounds, or compound combinations, for patients with NSCLC with different mutational backgrounds and disease stages.

Limitations of the study

PDTO establishment and TIL isolation success rates were modest (52.9% and 41.17%, respectively), which may limit the applicability of these models to certain patient samples. Our co-culture systems assess individual components of the TIME rather than fully recapitulating the complex, multi-cellular interactions of the native tumor microenvironment. While TAM reconstitution captures key aspects of myeloid involvement, other important cellular players, such as neutrophils, dendritic cells, and cancer-associated fibroblasts, are not included in the current models. Additionally, our study utilized treatment-naive tumor samples, and evaluating the feasibility and relevance of these co-culture systems using tumors from patients who have received therapy will be an important next step to assess their clinical utility and predictive value across treatment contexts. Finally, validation in tumor types beyond NSCLC is necessary to determine the broader applicability of these approaches.

RESOURCE AVAILABILITY

Lead contact

Further information and requests for resources and reagents should be directed to and will be fulfilled by the lead contact, M. Laura Martin (mlm4001@med.cornell.edu).

Materials availability

This study did not generate any new, unique reagents.

Model access is subject to scientific review and completion of a material transfer agreement through EIPM. Please see also https://www.cognitoforms.com/IPM3/EIPMCollaborationRequest.

Data and code availability

- All data reported in this manuscript, including PDTO and matching tumor genomics and macrophage transcriptomics, are available at dbGaP (Study Accession: phs004616.v1.p1).
- This manuscript does not report any original code.
- Any additional information required to reanalyze the data reported in this paper is available from the lead contact upon request.

ACKNOWLEDGMENTS

This work was supported by a Research Alliance between Eli Lilly and Company and the Englander Institute for Precision Medicine. Project support for this research was also provided, in part, by the Center for Translational Pathology from the Department of Pathology and Laboratory Medicine at Weill Cornell Medicine. J.M.M., M.S., J. Moyer., and M.A.A. are supported by the Department of Defense Prostate Cancer Research Program (PCRP) Health Disparity Research Award (PC200267).

AUTHOR CONTRIBUTIONS

Conceptualization, E.P., M.L.M., and O.E.; investigation, E.P., J.C., H.-H.K., M.A.A., G.M., M.V.R., J.N., A.I., H.R., S.A., T.K., J. Manohar., A.D.-L., M.S., J. Moyer., B.B., P.C., and M.M.; formal analysis, E.P. and M.L.M.; validation, E.P., M.L.M., O.E., K.B., J.M.M., V.M., A.S., H.G., and N.A.; writing – original draft, E.P. and M.L.M.; writing – review & editing, O.E., M.L.M., and E.P.; supervision, M.L.M. All authors read and approved the final manuscript.

DECLARATION OF INTERESTS

K.B. is a current employee and shareholder of Eli Lilly and Company. O.E. is an equity holder in or paid advisor to OneThree Bio, Owkin, Freenome, Champions Oncology, Pionyr Immunotherapeutics, Harmonic Discovery, Acuamark, and Genetic Intelligence. E.P. is a current employee of Pathos AI. M.L.M. is a current employee of Altos Labs. H.G. is currently an employee at The Ohio State University.

STAR★METHODS

Detailed methods are provided in the online version of this paper and include the following:

- KEY RESOURCES TABLE

CellPress
OPEN ACCESS

- EXPERIMENTAL MODEL AND STUDY PARTICIPANT DETAILS
  - Culture media formulations
  - Patient samples
- METHOD DETAILS
  - NSCLC-PDTO establishment and TIL isolation
  - T cell rapid expansion protocol
  - NSCLC-PDTOs histopathological and genomic characterization
  - Prediction of neoantigenic mutations
  - T cell and NSCLC-PDTOs co-culture
  - Quantification of IFNγ production by effector $CD8^+$ T-cells
  - PDTOs killing assay
  - PDTOs drug sensitivity assays
  - Tumor slides imaging mass-cytometry
  - Macrophage differentiation and polarization
  - Macrophage polarization assessment by bulk RNAseq
  - Macrophage phenotypic characterization by flow cytometry
- QUANTIFICATION AND STATISTICAL ANALYSIS

SUPPLEMENTAL INFORMATION

Supplemental information can be found online at https://doi.org/10.1016/j.crmeth.2026.101339.

Received: April 10, 2025
Revised: August 19, 2025
Accepted: February 6, 2026
Published: May 18, 2026

REFERENCES

1. Ganti, A.K., Klein, A.B., Cotarla, I., Seal, B., and Chou, E. (2021). Update of incidence, prevalence, survival, and initial treatment in patients with non-small cell lung cancer in the US. JAMA Oncol. *7*, 1824–1832.
2. Reck, M., Popat, S., Reinmuth, N., De Ruysscher, D., Kerr, K.M., and Peters, S.; ESMO Guidelines Working Group (2014). Metastatic non-small-cell lung cancer (NSCLC): ESMO clinical practice guidelines for diagnosis, treatment and follow-up. Ann. Oncol. *25*, iii27–iii39.
3. Zarogoulidis, K., Zarogoulidis, P., Darwiche, K., Boutsikou, E., Machairiotis, N., Tsakiridis, K., Katsikogiannis, N., Kougioumtzi, I., Karapantzos, I., Huang, H., and Spyratos, D. (2013). Treatment of non-small cell lung cancer (NSCLC). J. Thorac. Dis. *5*, S389–S396.
4. Ghosh, D.D., McDonald, H., Dutta, R., Krishnan, K., Thilakan, J., Paul, M.K., Arya, N., Rao, M., and Rangnekar, V.M. (2024). Prognostic indicators for precision treatment of non-small cell lung carcinoma. Cells *13*, 1785.
5. Pilotto, S., Molina-Vila, M.A., Karachaliou, N., Carbognin, L., Viteri, S., González-Cao, M., Bria, E., Tortora, G., and Rosell, R. (2015). Integrating the molecular background of targeted therapy and immunotherapy in lung cancer: a way to explore the impact of mutational landscape on tumor immunogenicity. Transl. Lung Cancer Res. *4*, 721–727.
6. Wu, Y., Yu, G., Jin, K., and Qian, J. (2024). Advancing non-small cell lung cancer treatment: the power of combination immunotherapies. Front. Immunol. *15*, 1349502.
7. Moya-Horno, I., Viteri, S., Karachaliou, N., and Rosell, R. (2018). Combination of immunotherapy with targeted therapies in advanced non-small cell lung cancer (NSCLC). Ther. Adv. Med. Oncol. *10*, 1758834017745012.
8. Rittmeyer, A., Barlesi, F., Waterkamp, D., Park, K., Ciardiello, F., von Pawel, J., Gadgeel, S.M., Hida, T., Kowalski, D.M., Dols, M.C., et al. (2017). Atezolizumab versus docetaxel in patients with previously treated non-small-cell lung cancer (OAK): a phase 3, open-label, multicentre randomised controlled trial. Lancet *389*, 255–265.
9. Santarpia, M., Gil, N., and Rosell, R. (2015). Strategies to overcome resistance to tyrosine kinase inhibitors in non-small-cell lung cancer. Expert Rev. Clin. Pharmacol. *8*, 461–477.
10. Wu, J., and Lin, Z. (2022). Non-small cell lung cancer targeted therapy: drugs and mechanisms of drug resistance. Int. J. Mol. Sci. *23*, 15056.
11. Genova, C., Dellepiane, C., Carrega, P., Sommariva, S., Ferlazzo, G., Pronzato, P., Gangemi, R., Filaci, G., Coco, S., and Croce, M. (2021). Therapeutic implications of tumor microenvironment in lung cancer: focus on immune checkpoint blockade. Front. Immunol. *12*, 799455.
12. Chong, C.R., and Jänne, P.A. (2013). The quest to overcome resistance to EGFR-targeted therapies in cancer. Nat. Med. *19*, 1389–1400.
13. Hummelink, K., van der Noort, V., Muller, M., Schouten, R.D., Lalezari, F., Peters, D., Theelen, W.S.M.E., Koelzer, V.H., Mertz, K.D., Zippelius, A., et al. (2022). PD-1T TILs as a predictive biomarker for clinical benefit to PD-1 blockade in patients with advanced NSCLC. Clin. Cancer Res. *28*, 4893–4906.
14. Rakaee, M., Adib, E., Ricciuti, B., Sholl, L.M., Shi, W., Alessi, J.V., Cortellini, A., Fulgenzi, C.A.M., Viola, P., Pinato, D.J., et al. (2023). Association of machine learning-based assessment of tumor-infiltrating lymphocytes on standard histologic images with outcomes of immunotherapy in patients with NSCLC. JAMA Oncol. *9*, 51–60.
15. Zhu, R., Huang, J., and Qian, F. (2025). The role of tumor-associated macrophages in lung cancer. Front. Immunol. *16*, 1556209.
16. Zheng, X., Weigert, A., Reu, S., Guenther, S., Mansouri, S., Bassaly, B., Gattenlöhner, S., Grimminger, F., Pullamsetti, S., Seeger, W., et al. (2020). Spatial density and distribution of tumor-associated macrophages predict survival in non-small cell lung carcinoma. Cancer Res. *80*, 4414–4425.
17. Pauli, C., Hopkins, B.D., Prandi, D., Shaw, R., Fedrizzi, T., Sboner, A., Sailer, V., Augello, M., Puca, L., Rosati, R., et al. (2017). Personalized in vitro and in vivo cancer models to guide precision medicine. Cancer Discov. *7*, 462–477.
18. Tiriac, H., Belleau, P., Engle, D.D., Plenker, D., Deschênes, A., Somerville, T.D.D., Froeling, F.E.M., Burkhart, R.A., Denroche, R.E., Jang, G.H., et al. (2018). Organoid profiling identifies common responders to chemotherapy in pancreatic cancer. Cancer Discov. *8*, 1112–1129.
19. Yao, Y., Xu, X., Yang, L., Zhu, J., Wan, J., Shen, L., Xia, F., Fu, G., Deng, Y., Pan, M., et al. (2020). Patient-derived organoids predict chemoradiation responses of locally advanced rectal cancer. Cell Stem Cell *26*, 17–26.e6.
20. Ooft, S.N., Weeber, F., Dijkstra, K.K., McLean, C.M., Kaing, S., van Werkhoven, E., Schipper, L., Hoes, L., Vis, D.J., van de Haar, J., et al. (2019). Patient-derived organoids can predict response to chemotherapy in metastatic colorectal cancer patients. Sci. Transl. Med. *11*, eaay2574.
21. Vlachogiannis, G., Hedayat, S., Vatsiou, A., Jamin, Y., Fernández-Mateos, J., Khan, K., Lampis, A., Eason, K., Huntingford, I., Burke, R., et al. (2018). Patient-derived organoids model treatment response of metastatic gastrointestinal cancers. Science *359*, 920–926.
22. Driehuis, E., Kretzschmar, K., and Clevers, H. (2021). Author correction: establishment of patient-derived cancer organoids for drug-screening applications. Nat. Protoc. *16*, 5739.
23. Neal, J.T., Li, X., Zhu, J., Giangarra, V., Grzeskowiak, C.L., Ju, J., Liu, I.H., Chiou, S.H., Salahudeen, A.A., Smith, A.R., et al. (2018). Organoid modeling of the tumor immune microenvironment. Cell *175*, 1972–1988.e16.
24. Forsythe, S.D., Erali, R.A., Laney, P., Sivakumar, H., Li, W., Skardal, A., Soker, S., and Votanopoulos, K.I. (2022). Application of immune enhanced organoids in modeling personalized Merkel cell carcinoma research. Sci. Rep. *12*, 13865.
25. Homicsko, K. (2020). Organoid technology and applications in cancer immunotherapy and precision medicine. Curr. Opin. Biotechnol. *65*, 242–247.
26. Dijkstra, K.K., Cattaneo, C.M., Weeber, F., Chalabi, M., van de Haar, J., Fanchi, L.F., Slagter, M., van der Velden, D.L., Kaing, S., Kelderman, S., et al. (2018). Generation of tumor-reactive T cells by co-culture of

peripheral blood lymphocytes and tumor organoids. Cell *174*, 1586–1598.e12.

27. Chauvat, A., Benhamouda, N., Gey, A., Lemoine, F.M., Paulie, S., Carrat, F., Gougeon, M.L., Rozenberg, F., Krivine, A., Cherai, M., et al. (2014). Clinical validation of IFNgamma/IL-10 and IFNgamma/IL-2 FluoroSpot assays for the detection of Tr1 T cells and influenza vaccine monitoring in humans. Hum. Vaccin. Immunother. *10*, 104–113.
28. Skoulidis, F., Byers, L.A., Diao, L., Papadimitrakopoulou, V.A., Tong, P., Izzo, J., Behrens, C., Kadara, H., Parra, E.R., Canales, J.R., et al. (2015). Co-occurring genomic alterations define major subsets of KRAS-mutant lung adenocarcinoma with distinct biology, immune profiles, and therapeutic vulnerabilities. Cancer Discov. *5*, 860–877.
29. Sedighzadeh, S.S., Khoshbin, A.P., Razi, S., Keshavarz-Fathi, M., and Rezaei, N. (2021). A narrative review of tumor-associated macrophages in lung cancer: regulation of macrophage polarization and therapeutic implications. Transl. Lung Cancer Res. *10*, 1889–1916.
30. Sachs, N., de Ligt, J., Kopper, O., Gogola, E., Bounova, G., Weeber, F., Balgobind, A.V., Wind, K., Gracanin, A., Begthel, H., et al. (2018). A living biobank of breast cancer organoids captures disease heterogeneity. Cell *172*, 373–386.e10.
31. Verduin, M., Hoeben, A., De Ruysscher, D., and Vooijs, M. (2021). Patient-derived cancer organoids as predictors of treatment response. Front. Oncol. *11*, 641980.
32. Kim, S.Y., Kim, S.M., Lim, S., Lee, J.Y., Choi, S.J., Yang, S.D., Yun, M.R., Kim, C.G., Gu, S.R., Park, C., et al. (2021). Modeling clinical responses to targeted therapies by patient-derived organoids of advanced lung adenocarcinoma. Clin. Cancer Res. *27*, 4397–4409.
33. Wang, S., Sun, J., Chen, K., Ma, P., Lei, Q., Xing, S., Cao, Z., Sun, S., Yu, Z., Liu, Y., and Li, N. (2021). Perspectives of tumor-infiltrating lymphocyte treatment in solid tumors. BMC Med. *19*, 140.
34. Lopez de Rodas, M., Nagineni, V., Ravi, A., Datar, I.J., Mino-Kenudson, M., Corredor, G., Barrera, C., Behlman, L., Rimm, D.L., Herbst, R.S., et al. (2022). Role of tumor infiltrating lymphocytes and spatial immune heterogeneity in sensitivity to PD-1 axis blockers in non-small cell lung cancer. J. Immunother. Cancer *10*, e004440.
35. Huang, J., Khong, H.T., Dudley, M.E., El-Gamil, M., Li, Y.F., Rosenberg, S.A., and Robbins, P.F. (2005). Survival, persistence, and progressive differentiation of adoptively transferred tumor-reactive T cells associated with tumor regression. J. Immunother. *28*, 258–267.
36. Powell, D.J., Jr., Dudley, M.E., Robbins, P.F., and Rosenberg, S.A. (2005). Transition of late-stage effector T cells to CD27+ CD28+ tumor-reactive effector memory T cells in humans after adoptive cell transfer therapy. Blood *105*, 241–250.
37. Langer, C.J., Gadgeel, S.M., Borghaei, H., Papadimitrakopoulou, V.A., Patnaik, A., Powell, S.F., Gentzler, R.D., Martins, R.G., Stevenson, J.P., Jalal, S.I., et al. (2016). Carboplatin and pemetrexed with or without pembrolizumab for advanced, non-squamous non-small-cell lung cancer: a randomised, phase 2 cohort of the open-label KEYNOTE-021 study. Lancet Oncol. *17*, 1497–1508.
38. Tran, K.Q., Zhou, J., Durflinger, K.H., Langhan, M.M., Shelton, T.E., Wunderlich, J.R., Robbins, P.F., Rosenberg, S.A., and Dudley, M.E. (2008). Minimally cultured tumor-infiltrating lymphocytes display optimal characteristics for adoptive cell therapy. J. Immunother. *31*, 742–751.
39. Baitsch, L., Legat, A., Barba, L., Fuertes Marraco, S.A., Rivals, J.P., Baumgaertner, P., Christiansen-Jucht, C., Bouzourene, H., Rimoldi, D., Pircher, H., et al. (2012). Extended co-expression of inhibitory receptors by human CD8 T-cells depending on differentiation, antigen-specificity and anatomical localization. PLoS One *7*, e30852.
40. Blackburn, S.D., Shin, H., Haining, W.N., Zou, T., Workman, C.J., Polley, A., Betts, M.R., Freeman, G.J., Vignali, D.A.A., and Wherry, E.J. (2009). Coregulation of CD8+ T cell exhaustion by multiple inhibitory receptors during chronic viral infection. Nat. Immunol. *10*, 29–37.
41. Drake, C.G. (2015). Combined immune checkpoint blockade. Semin. Oncol. *42*, 656–662.
42. Phan, T.G., Long, G.V., and Scolyer, R.A. (2015). Checkpoint inhibitors for cancer immunotherapy: multiple checkpoints on the long road towards cancer immunotherapy. Immunol. Cell Biol. *93*, 323–325.
43. Martinez, M., Kim, S., St Jean, N., O'Brien, S., Lian, L., Sun, J., Verona, R.I., and Moon, E. (2021). Addition of anti-TIM3 or anti-TIGIT antibodies to anti-PD1 blockade augments human T cell adoptive cell transfer. OncoImmunology *10*, 1873607.
44. Shaw, A.T., and Engelman, J.A. (2013). ALK in lung cancer: past, present, and future. J. Clin. Oncol. *31*, 1105–1111.
45. Zhu, S., Ma, A.H., Zhu, Z., Adib, E., Rao, T., Li, N., Ni, K., Chittepu, V.C.S.R., Prabhala, R., Garisto Risco, J., et al. (2021). Synergistic antitumor activity of pan-PI3K inhibition and immune checkpoint blockade in bladder cancer. J. Immunother. Cancer *9*, e002917.
46. Sun, P., and Meng, L.H. (2020). Emerging roles of class I PI3K inhibitors in modulating tumor microenvironment and immunity. Acta Pharmacol. Sin. *41*, 1395–1402.
47. Cullis, J., Das, S., and Bar-Sagi, D. (2018). Kras and tumor immunity: friend or foe? Cold Spring Harb. Perspect. Med. *8*, a031849.
48. Hamarsheh, S., Groß, O., Brummer, T., and Zeiser, R. (2020). Immune modulatory effects of oncogenic KRAS in cancer. Nat. Commun. *11*, 5439.
49. Zhu, Y.M., Webster, S.J., Flower, D., and Woll, P.J. (2004). Interleukin-8/CXCL8 is a growth factor for human lung cancer cells. Br. J. Cancer *91*, 1970–1976.
50. Kang, P., Liu, D., Li, L., Guo, X., Ye, Y., Li, Y., Jiang, Q., Lin, S., and Yuan, Q. (2023). Interleukin 8 in plasma is an efficacy marker for advanced non-small cell lung cancer treated with hypofractionated radiotherapy and PD-1 blockade. Cytokine *163*, 156133.
51. Huseni, M.A., Wang, L., Klementowicz, J.E., Yuen, K., Breart, B., Orr, C., Liu, L.F., Li, Y., Gupta, V., Li, C., et al. (2023). CD8(+) T cell-intrinsic IL-6 signaling promotes resistance to anti-PD-L1 immunotherapy. Cell Rep. Med. *4*, 100878.
52. Yan, X., Orentas, R.J., and Johnson, B.D. (2006). Tumor-derived macrophage migration inhibitory factor (MIF) inhibits T lymphocyte activation. Cytokine *33*, 188–198.
53. Pan, Y., Yu, Y., Wang, X., and Zhang, T. (2020). Tumor-associated macrophages in tumor immunity. Front. Immunol. *11*, 583084.
54. Zhang, Q., and Sioud, M. (2023). Tumor-associated macrophage subsets: shaping polarization and targeting. Int. J. Mol. Sci. *24*, 7493.
55. Ho, C.C., Liao, W.Y., Wang, C.Y., Lu, Y.H., Huang, H.Y., Chen, H.Y., Chan, W.K., Chen, H.W., and Yang, P.C. (2008). TREM-1 expression in tumor-associated macrophages and clinical outcome in lung cancer. Am. J. Respir. Crit. Care Med. *177*, 763–770.
56. Balazova, K., Clevers, H., and Dost, A.F.M. (2023). The role of macrophages in non-small cell lung cancer and advancements in 3D co-cultures. eLife *12*.
57. Liu, J., Zhang, B., Cui, Y., Song, H., and Shang, D. (2024). In vitro co-culture models for studying organoids-macrophages interaction: the golden technology of cancer immunotherapy. Am. J. Cancer Res. *14*, 3222–3240.
58. Jiang, S., Deng, T., Cheng, H., Liu, W., Shi, D., Yuan, J., He, Z., Wang, W., Chen, B., Ma, L., et al. (2023). Macrophage-organoid co-culture model for identifying treatment strategies against macrophage-related gemcitabine resistance. J. Exp. Clin. Cancer Res. *42*, 199.
59. Jin, J., Sabatino, M., Somerville, R., Wilson, J.R., Dudley, M.E., Stroncek, D.F., and Rosenberg, S.A. (2012). Simplified method of the growth of human tumor infiltrating lymphocytes in gas-permeable flasks to numbers needed for patient treatment. J. Immunother. *35*, 283–292.
60. Pauli, C., Puca, L., Mosquera, J.M., Robinson, B.D., Beltran, H., Rubin, M.A., and Rao, R.A. (2016). An emerging role for cytopathology in precision oncology. Cancer Cytopathol. *124*, 167–173.

CellPress
OPEN ACCESS

61. Sailer, V., Pauli, C., Merzier, E.C., Mosquera, J.M., Beltran, H., Rubin, M.A., and Rao, R.A. (2017). On-site cytology for development of patient-derived three-dimensional organoid cultures: a pilot study. Anticancer Res. *37*, 1569–1573.

62. Cancer Genome Atlas Research Network (2014). Comprehensive molecular profiling of lung adenocarcinoma. Nature *511*, 543–550.

63. Hundal, J., Kiwala, S., McMichael, J., Miller, C.A., Xia, H., Wollam, A.T., Liu, C.J., Zhao, S., Feng, Y.Y., Graubert, A.P., et al. (2020). pVACtools: a computational toolkit to identify and visualize cancer neoantigens. Cancer Immunol. Res. *8*, 409–420.

64. Yamashita, S. (2007). Heat-induced antigen retrieval: mechanisms and application to histochemistry. Prog. Histochem. Cytochem. *41*, 141–200.

STAR★METHODS

KEY RESOURCES TABLE

REAGENT or RESOURCE	SOURCE	IDENTIFIER
Antibodies		
Mouse-anti human CD3 (OKT3)	Biolegend	317302: RRID: AB_571927
Mouse-anti human PD-1	BioXcell	SIM0010: RRID: AB_2894731
Mouse-anti human PD-1 (LSN3415244)	Eli Lilly	provided by Eli lilly
Mouse-anti human TIM3 (F38-2E2)	eBioscience	16-3109-85: RRID: AB_2573083
Mouse-anti human TIM3 (LY3321367)	Eli Lilly	provided by Eli lilly
Mouse-anti human LAG3 (BLR027F)	invitrogen	MA5-44249: RRID: AB_2926379
Mouse-anti human TIGIT	BPS Bioscience	71340: RRID: AB_2742063
Mouse-anti human PDL1(LY3300054)	Eli Lilly	provided by Eli lilly
Mouse-anti human PD-1/PDL1 (LY3434172)	Eli Lilly	provided by Eli lilly
Mouse-anti human CD3 BV605	Biolegend	300460: RRID: AB_2564380
Mouse-anti human CD4 BV711	Biolegend	317440: RRID: AB_2562912
Mouse-anti human CD8a BV785	Biolegend	301046: RRID: AB_2563264
Mouse-anti human TNFa PerCp- Cy5.5	Biolegend	502926: RRID: AB_2204081
Mouse-anti human IFNg PE	Biolegend	502509: RRID: AB_315234
Mouse-anti human PD-1 PE-Cy7	Biolegend	329918: RRID: AB_2159324
Mouse-anti human PD-1- Alexa 700	Biolegend	329952: RRID: AB_2566364
Mouse-anti human TIM3-FITC	Biolegend	345022: RRID: AB_2563937
Mouse-anti human LAG-3-PE	Biolegend	369306: RRID: AB_2629592
Mouse-anti human TIGIT-APC	Biolegend	372706: RRID: AB_2632732
Mouse-anti human PD-1 BV421	Biolegend	329920: RRID: AB_10960742
Mouse-anti human CD8 PerCp-Cy5.5	Biolegend	344710: RRID: AB_2044010
Mouse-anti human CD3 APC-CY7	Biolegend	300318: RRID: AB_314054
Mouse-anti human CD4-APC	Biolegend	344614: RRID: AB_2028488
Mouse-anti human TIGIT-BV605	Biolegend	372712: RRID: AB_2632927
Mouse-anti human MHCII-FITC	Biolegend	361706: RRID: AB_2563192
Mouse-anti human CEACAM1-PE	Biolegend	342304: RRID: AB_2077337
Mouse-anti human LSECtin-APC	RyD	FAB2947A: RRID: AB_3648900
Mouse-anti human NECTIN-2/CD112-Percp-Cy5.5	Biolegend	337416: RRID: AB_2565734
Mouse-anti human CD155-BV421	Biolegend	337631: RRID: AB_2810525
Mouse-anti human CD206- FITC	Biolegend	321104: RRID: AB_571905
Mouse-anti human CD163 BV711	Biolegend	333630: RRID: AB_2650972
Mouse-anti human HLA-DR BV605	Biolegend	365604: RRID: AB_3083355
Mouse-anti human CD80 BV421	Biolegend	305222: RRID: AB_2564407
Mouse-anti human TREM-1-APC	Biolegend	314909: RRID: AB_10644181
Mouse-anti human MARCO-PE	eBioscience	12-5447-42: RRID: AB_2762430
Mouse-anti human CD24 PerCp-Cy5.5	Biolegend	311116: RRID: AB_10960741
Mouse-anti human CD66e/c	Biolegend	364902: RRID: AB_2904391
Mouse-anti human HLA-ABC-PE	BD	557349: RRID: AB_396655
Mouse-anti human CEACAM1-PE	Biolegend	342304: RRID: AB_2077337
Mouse-anti human NECTIN-2/CD112-PE	Biolegend	337409: RRID: AB_2174163
Mouse-anti human CD155-PE	Biolegend	337609: RRID: AB_2253258
Mouse-anti human PDL1-PE	Biolegend	329705: RRID: AB_940366
Mouse-anti human CD155	CST	31235SF: RRID: AB_2741378

(*Continued on next page*)

CellPress
OPEN ACCESS

Continued

REAGENT or RESOURCE	SOURCE	IDENTIFIER
Mouse-anti human CD3	Fluidigm	3170019D: RRID: AB_2811048
Mouse-anti human CD8a	BioLegend	372902: RRID: AB_2650657
Mouse-anti human CEACAM1	R&D	MAB22441: RRID: AB_2077346
Mouse-anti human HLA_ABC	Abcam	ab70328: RRID: AB_1269092
Mouse-anti human IFNg	Abcam	ab218890: RRID: AB_2847937
Mouse-anti human Nectin-2/CD112	Abcam	ab239346: RRID: AB_447553
Mouse-anti human PD-1	CST	63815SF: RRID: AB_2728819
Mouse-anti human PD-L1	Abcam	ab236238: RRID: AB_2832197
Mouse-anti human TIGIT	Abcam	ab243903: RRID: AB_2943164
Mouse-anti human TIM3	CST	81229SF: RRID: AB_2716862
Mouse-anti human IgG1-PerCp-Cy5.5	Biolegend	400150: RRID: AB_893664
Mouse-anti human IgG1-PE	Biolegend	400112: RRID: AB_2847829
Mouse-anti human IgG1-PE-Cy7	Biolegend	400126: RRID: AB_326448
Mouse-anti human IgG1-APC	Biolegend	400120: RRID: AB_871704
Mouse-anti human IgG1- APC-Cy7	BD bio	557873: RRID: AB_396915
Mouse-anti human IgG1- FITC	BD bio	555748: RRID: AB_396090
Mouse-anti human IgG1- Alexa 488	Biolegend	406626: RRID: AB_2715989
Mouse-anti human IgG1- BV421	Biolegend	400158: RRID: AB_11150232
Mouse-anti human IgG2a-BV605	Biolegend	400270: RRID: AB_3097669
Mouse-anti human IgG2a-APC	Biolegend	400220: RRID: AB_11044786
Mouse-anti human IgG1-Alexa 700	Biolegend	400144: RRID: AB_10972478
Mouse-anti human IgG2a- PerCp-Cy5.5	Biolegend	400257: RRID: AB_470216
Mouse-anti human IgG2b- Pacific Blue	Biolegend	400331: RRID: AB_795864
Mouse-anti human IgG2a-BV711	Biolegend	400272: RRID: AB_3097679
Mouse-anti human IgG2A- FITC	Biolegend	400210: RRID: AB_326458
Mouse-anti human IgG1-Alexa750	RD	IC002S: RRID: AB_3654368
Mouse-anti human IgG2b-PE	Biolegend	401208: RRID: AB_11043548
**Chemicals, peptides, and recombinant proteins**		
Human recombinant IL-2	Peprotech	200–02
Zombie Violet Fixable viability kit	Biolegend	423114
Zombie UV Fixable viability kit	Biolegend	423108
Cell trace Far red	Thermo Fisher Scientific	34564
NucView 488 caspase-3 substrate	Biotium	10402
TrypLE Express Enzyme 1x	Gibco	12604021
Ficoll-paque Plus	Millipore sigma	GE-17-1440-02
Cell recovery solution	Corning	354253
DAPI	Thermo Fisher Scientific	D1306
Ionomycin	Sigma-Aldrich	I9657
PMA	Sigma-Aldrich	16561
Human recombinant IFNγ	peprotech	300–02
Human recombinant IL-10	peprotech	200–10
Human recombinant IL-4	peprotech	200–04
Human recombinant TGFβ	peprotech	100–21
Human recombinant LIF	peprotech	300–05
Adenosine	Sigma-Aldrich	A4036
Human recombinant GM-CSF	peprotech	300–03
Human recombinant M-CSF	peprotech	300–25
Lipopolysaccharide (LPS)	Thermo Fisher Scientific	00-4976-93

(*Continued on next page*)

Continued

REAGENT or RESOURCE	SOURCE	IDENTIFIER
Cell trace Blue	Thermo Fisher Scientific	34568
NucGreen™ Dead 488 ReadyProbes™ Reagent	Thermo Fisher Scientific	37109
RPMI 1460	Gibco	11-875-119
10% FBS	Gibco	F2442
2mM Glutamax	Thermo Fisher Scientific	35050079
100 U/mL Penicilin/Streptomycin	Gibco	11548876
25 mM HEPES	Gibco	15630080
10% Human AB Serum	Sigma-Aldrich	H4522
Advance DMEM	Gibco	12491015
100μg/ml Primocin	Invivogen	NC9141851
10 μmol/L Rock inhibitor Y-27632	Selleckchem.com	S1049
250 U/ml Collagenase IV	Thermo Fisher Scientific	17104019
1mg/ml Collagenase I	Worthington Biochemical	LS004196
1X B27	Gibco	17504044
10% noggin conditioned media	In house	In house
10% R-spondin conditioned media	In house	In house
10 mM Nicotinamide	Sigma-Aldrich	98-92-0
1.25 mM N-acetylcysteine	Sigma-Aldrich	616-91-1
1ng/ml Recombinant Human FGF-b	Peprotech	100-18B
20ng/mL Recombinant Human FGF-10	Peprotech	100–26
1μM PGE2	biotechne- Tocris	363-24-6
10 μM SB202190	Sigma-Aldrich	152121-30-7
50ng/mL Mouse Recombinant EGF	Gibco	PMG-8041
10ng/mL Heregulin Beta-1	Peprotech	100–03
500nM A-83-01	biotechne- Tocris	909910-43-6
Lapatinib	MedChem Express	HY-50898
Osimertinib	MedChem Express	HY-15772
Dacomitinib	Selleckchem	S2727
Afatinib	MedChem Express	HY-10261B
Erlotinib	Selleckchem	S7786
Gefitinib	MedChem Express	HY-50895
Idelalisib	MedChem Express	HY-13026
Copanlisib dihydrochloride	MedChem Express	HY-15346A
PI-103	Selleckchem	S1038
Buparlisib	Selleckchem	S2247
GSK2636771	MedChem Express	HY-15245
Parsaclisib	MedChem Express	HY-109068
Erganelisib	MedChem Express	HY-100716
Temsirolimus	MedChem Express	HY-50910
Rapamycin	MedChem Express	HY-10219
Everolimus	MedChem Express	HY-10218
Ulixertinib	Selleckchem	S7854
AZ628	Selleckchem	S2746
Dabrafenib Mesylate	MedChem Express	HY-14660A
AMG510	MedChem Express	HY-114277
Binimetinib	MedChem Express	HY-15202
Trametinib	MedChem Express	HY-10999
Selumetinib	MedChem Express	HY-50706

(*Continued on next page*)

Continued

REAGENT or RESOURCE	SOURCE	IDENTIFIER
Gemcitabine	MedChem Express	HY-17026
Carboplatin	MedChem Express	HY-17393
Paclitaxel	MedChem Express	HY-B0015
Critical commercial assays		
FluoroSpot Plus: Human IFN-γ/Granzyme B/IL-2	Mabtech	FSP-013602-2
BD Fixation and permeabilization Kit	BD	554714
CD14 Microbeads, Human	Miltenyi Biotech	130-050-201
Mojo sort human CD8 T cell Isolation kit	Biolegend	480129
Proteome Profiler Human XL Cytokine Array Kit	R&D Systems	ARY022B
CellTiter-Glo 3D	Promega	G9681
Experimental models: Cell lines		
WCM2499	*Ex vivo* models platform- EIPM-Weill Cornell Medicine	N/A
WCM3407	*Ex vivo* models platform- EIPM-Weill Cornell Medicine	N/A
WCM3606	*Ex vivo* models platform- EIPM-Weill Cornell Medicine	N/A
WCM3417	*Ex vivo* models platform- EIPM-Weill Cornell Medicine	N/A
WCM3409	*Ex vivo* models platform- EIPM-Weill Cornell Medicine	N/A
WCM3410	*Ex vivo* models platform- EIPM-Weill Cornell Medicine	N/A
WCM3413	*Ex vivo* models platform- EIPM-Weill Cornell Medicine	N/A
WCM3416	*Ex vivo* models platform- EIPM-Weill Cornell Medicine	N/A
WCM3289	*Ex vivo* models platform- EIPM-Weill Cornell Medicine	N/A
Deposited data		
Whole exome sequencing (WES), Oncomine/TruSight Oncology (TSO) 500 targeted sequencing of PDTOs and matching tumors	This paper	phs004616.v1.p1
RNAseq of Macrophages	This paper	phs004616.v1.p1

EXPERIMENTAL MODEL AND STUDY PARTICIPANT DETAILS

Culture media formulations

Culture media formulations are shown in Table S4 and key resources table.

Patient samples

Tissue and whole-blood specimens were obtained from 17 patients with lung cancer diagnosis that underwent surgical procedures between November 2020 and June 2022. Specimens were obtained in accordance with New York Presbyterian hospital- Weill Cornell Medical College (NYPH-WCM) guidelines and under an Institutional Review Board approved protocol (IRB #1008011221). The cohort included 9 females (52.9%) and 8 males (47.1%), with racial/ethnic distribution as follows: White (n = 13, 76.5%), African American (n = 2, 11.8%), Asian (n = 2, 11.8%), and unknown (n = 1, 5.9%). Smoking status was distributed among never smokers (n = 7, 41.2%), former smokers (n = 7, 41.2%), and current smokers (n = 3, 17.6%). Cancer stages ranged from early (IA2, IA3, IB) to advanced disease (IIIA, IIIB, IVA), with TNM staging showing predominantly T1-T4 primary tumors, N0-N2 nodal involvement, and M0-M1a distant metastasis status. The majority of patients presented with no lymph node involvement (N0: n = 12, 70.6%) and no distant metastases (M0: n = 15, 88.2%). Age data were not available for this cohort, which represents a limitation in characterizing the complete demographic profile of this patient population. We only included tumors that were larger than 2cm. Once

resected, tumor samples were placed on transport media and kept on ice until processing (within 5 h after resection). A small fraction of the tumor resection was placed in formalin for subsequent histopathological review. PBMC samples were retrieved from the NYPH-WCM thoracic surgery biobank at the moment of the co-culture experiments.

METHOD DETAILS

NSCLC-PDTO establishment and TIL isolation

When received, tumor resections were split in two pieces. One-half was processed for PDTO establishment and the other for TIL isolation.

PDTOs were developed as previously described by Pauli et al. with modifications.[17] Fresh tissue samples were washed three times with transport media and placed in a sterile 3-cm Petri dish for mechanical dissection into smaller pieces (2mm diameter) prior to enzymatic digestion. Media containing loose cells or clumps of cells after mechanical dissection were separated from tissue pieces as a "pre-digest" fraction and used later for culture without enzymatic digestion. Enzymatic digestion was done with collagenase IV media in a volume of at least 20 times the tissue volume and incubated on a shaker at 200 rpm at 37°C until the digestion solution turned cloudy, typically 30–45 min. The suspension and the pre-digest fraction were both centrifuged at 300g for 3 min and the cell pellet was washed once with washing media. The cells in each fraction were resuspended separately in a small volume of PDTO culture media. Up to ten 100 μL drops of Matrigel/cell suspension were distributed into a 6-well cell suspension culture plate (Cellstar cat #657185). The drops were allowed to polymerize for 30 min inside the incubator at 37°C and 5% CO2 and afterward, 3 mL PDTO culture media were added per well. Fresh culture media was replaced every 3 to 4 days. PDTOs at approximately 300–500 μm were passaged using TrypLE Express for 10–12 min in the water bath at 37°C. Single cells and small cell clusters were replated according to the procedure described above. Monthly mycoplasma screening was performed using the abm Mycoplasma PCR Detection Kit (Cat# G238). PDTOs were cryopreserved in Recovery Cell Culture Freezing Medium (Cat# 12648010) in liquid nitrogen. For inquiries regarding PDTO model requests, please contact eipmfusion@med.cornell.edu.

For TIL isolation, samples were mechanically dissected, resuspended in collagenase I media and incubated for 30 min at 37°C in a humidified incubator. After that, digested tissue samples were chopped again and incubated in collagenase I media for another 30 min. After the incubation, digested tissue was chopped again, and cellular suspensions were filtered through 40μm cell strainers and centrifuged at 300g for 3 min. Cells were resuspended and incubated for 3 min in ACK lysis buffer (Quality biological cat #119-156-721) to eliminate contaminant erythrocytes, centrifugated again and resuspended at a concentration of $5x10^5$ cells/ml in T cell culture media supplemented with 3000u/ml IL-2 and 0.5 μg/mL anti-CD3 (OKT3 clone) to favor initial T cell proliferation. Half of the media was replaced every 3 days.

T cell rapid expansion protocol

Once T cell clusters were observed indicating that the cultures were stabilized, cells were harvested, and a rapid expansion protocol (REP) was performed to increase the number of available T-cells. REP was performed as previously described by Jin et al.[59] Briefly, stabilized TILs were cultured with irradiated allogeneic PBMCs (40 or 50Gy) at a ratio of 1:200 in T cell culture media supplemented with 3000u/ml IL-2 and 1 μg/mL anti-CD3 (OKT3 clone), refreshing half of the media every 3 days. After 10 days of culture, cells were harvested and cryopreserved on T cell freezing media.

NSCLC-PDTOs histopathological and genomic characterization

PDTOs histopathology was verified by comparing sections from formalin-fixed and paraffin-embedded (FFPE) passage 5 PDTOs blocks to parent tumor sections using our developed cytology and histology platforms.[60,61] Briefly, PDTOs were released from Matrigel droplets using cell recovery solution, suspended in a fibrinogen/thrombin gel pellet, fixed with 4% paraformaldehyde in PBS, and embedded in paraffin to create FFPE blocks. Hematoxylin and eosin (H&E) stained sections of the FFPE blocks were verified as tumor cells and compared to H&E-stained sections from the corresponding tumors to verify matching cellular morphology by a WCM pathologist. Whole exome sequencing (WES) or Oncomine/TruSight Oncology (TSO) 500 targeted sequencing was performed on PDTOs pellets from passage 5 and matching tumors to confirm identity and mutational profile concordance. Single nucleotide variants found in tumor and PDTOs samples via WES, Oncomine or TSO 500 were compared in order to verify concordance of driving mutations in matching samples. We addressed the mutational state and copy number alterations of *TP53, KRAS, KEAP1, STK11, EGFR, NF1, BRAF, SETD2, RBM10, MGA, MET, ARID1A, PIK3CA, SMARCA4, RB1, CDKN2A, U2AF1, RIT1, HER2* genes reported to be relevant drivers in NSCLC.[62]

Prediction of neoantigenic mutations

To identify potential neoantigens from non-synonymous and truncating mutations pVACtools were used.[63] Seven epitope prediction methods (MHCflurry, MHCnuggetsI, NetMHC, NetMHCpan, PickPocket, SMM, and SMMPMBEC) were applied to identify neoantigens restricted to patient specific MHC Class I alleles (HLA-A, -B, -C). For each prediction, method with best binding affinity (i.e., lowest IC50) was used to filter and prioritize neoantigens as follows: 1) Neoantigens with IC50 $\leq$ 1,000 μM, 2) Fold change between mutant vs. corresponding wild-type epitope $\geq$2, and 3) when available, gene expression value (FPKM) of >1.

T cell and NSCLC-PDTOs co-culture

As previously described by Djskstra et al.; tumor reactive T-cells were expanded by co-culturing them during 14 days with autologous PDTOs in T cell culture media supplemented with 300UI/ml of IL-2. T-cells were rechallenged at day 7 of co-culture and half of the medium was refreshed every 3 days. At day 14, T-cells were rechallenged with PDTOs and functional assays were performed. Different monoclonal antibodies (mAbs) provided by Eli Lily: anti-PD-1 (LSN3415244), anti-PDL1 (LY3300054), anti-TIM3 (LY3321367) and anti PD-1/PDL1 (LY3434172) were added at day 0, 7 and 14 of co-culture to address their impact on tumor specific-T cell expansion and effector function. Matching isotype huIgG was used as controls.

Quantification of IFNγ production by effector CD8$^+$ T-cells

Intracellular cytokine staining (ICS)

After 14 days of co-culture with PDTOs, T-cells were collected and seeded in round bottom 96-well plates. PDTOs were added in a 1:5 (PDTOs: effector cell) ratio. After 1 h of incubation, GolgiStop (BD) was added (to allow intracellular cytokines accumulation) for 4hs. After this incubation, cells were harvested and washed once with PBS. Anti-CD3, CD4 and CD8 surface stainings were performed by incubating cells with the respective antibody cocktail for 20 min. Cells were washed, stained with the fixable viability dye during 20 min at room temperature. Then, cells were washed, fixed and permeabilized with BD fix and perm kit following manufacturer instructions. After permeabilization, cells were incubated with anti-IFNγ for 20 min. Finally, cells were washed with PBS and seeded in FACs tubes for acquisition employing BD symphony A5 cytometer. Due to limited cell availability, ICS was performed without technical replicates. However, to enhance biological robustness, T-cells for each condition were divided into two separate wells (250.000–400.000 cells/well) and independently stimulated with PDTOs at a 1:5 ratio. Following incubation, cells from both wells were pooled prior to staining, ensuring that each reported measurement reflects the response of independently stimulated cell populations cultured under identical conditions.

FluoroSpot assay (FS)

T-cells were collected after 14 days of co-culture and samples were enriched on CD8$^+$ T-cells using the mojosort CD8$^+$ negative selection Kit following the manufacturer instructions. 100.000T-cells/well were seeded in pre-coated FS plates (mabtech FluoroSpot flex kit) in 200ul of T cell culture media and incubated overnight with PDTOs in a 1:5 ratio. As positive controls, T-cells were stimulated with Phorbol 12-myristate 13-acetate (25ng/ml) and Ionomycin (1μg/ml). To record basal detection levels, T-cells were seeded in T cell media alone. Each condition was run in triplicates. FS plates were developed following manufacturer instructions. Plates were sent to Zellnet Consulting for reading services (Mabtech IRIS). Average fold change in the number of spots (baseline/experimental condition) + SD and activity (spots intensity x spots size/1000) were reported.

PDTOs killing assay

T-cells recovered after 2 weeks of co-culture were rechallenged with PDTOs to assess their cytotoxic potential. 3 days before the tumor killing assay, 3-5 x 10^4 single tumor cells were stained with Cell trace far red and seeded in 100ul Matrigel 66% droplets. After 72hs far red stained PDTOs were harvested using cell recovery solution to preserve the 3D structure and seeded in 96 well plates in 150 μL of T cell media containing 5uM NucView488 caspase-3 substrate. T-cells were added in a 3:1 ratio (effector:target) in 50 μL of media. Plates were imaged every 1 h using Incucyte S3 (Sartorius) for 12 h recording 3 fields/well. The 12 h timepoint was selected based on several technical constraints observed during optimization. Beyond this timepoint, PDTOs harvested with cell recovery solution began dissociating into single cells, increasing baseline apoptosis and inter-replicate variability. Additionally, T cell media used during co-culture, while necessary to preserve T cell functions, lacks the complex supplements required for extended PDTOs viability. Finally, accumulation of cleaved NucView488 fluorescent fragments in the absence of phagocytes contributed to increased background signal at later timepoints. While these factors allowed reliable measurements up to 20 h for most of the patients, the earliest onset of elevated baseline apoptosis occurred at 14h in the most sensitive sample. To ensure consistency across all samples and maintain comparability between patients, we standardized data reporting at 12 h. A minimal threshold area of 50μm^2 was established for red events (PDTOs) to be quantified. Apoptotic PDTOs were identified as double-positive cells (green + red signal). Percentage of apoptotic PDTOs was calculated based on the total number of red events/field. Apoptotic percentage in each condition was determined using PDTOs treated with 10uM of staurosporine as 100% apoptosis.

PDTOs drug sensitivity assays

PDTOs were digested into a single cell suspension and cells were plated in a 384 well plate (Thermo Scientific Nunc cat#142761) at a density of 1000 cells per well in 8ul droplets (1:2 media:Matrigel). Plates were centrifuged briefly to ensure the cells were at the bottom of the well and 15μL of media were added. Cells were incubated for 72h to allow the cells to form PDTOs and afterward the drugs were added and incubated for additional 96h. The readout was performed using CellTiterGlo3D reagent according to the manufacturer's protocol. Luminescence was measured by the Biotek Synergy H4 plate reader.

To perform combination assays, we adapted our tumor killing assay: 800–1000 tumor cells were seeded in PDTO-media with 25% Matrigel on 384 wells plates and cultured for 4 days. At day 4, targeted agents were added and incubated for 3 days after which half of the media (15μL) was replaced with T cell media containing T-cells and the NucView488 caspase-3 substrate. Apoptotic PDTOs were quantified after 24hs of T cell addition.

Tumor slides imaging mass-cytometry

Banked human lung FFPE tissue samples were baked for 2 h on a slide warmer at 60^{0}C. The slides were then dewaxed in CitriSolv (Decon Labs, cat#1601) twice, each for 10 min, followed by hydration in descending series of ethanol (100%, 95%, 80% and 75%) for 5 min each. The slides were washed in Milli Q water and processed for antigen retrieval using Heat Induced Epitope Retrival method at 95^{0}C for 30 min.[64] The slides were then cooled to room temperature and washed twice in TBS. After blocking the slides for an hour in SuperBlock blocking buffer (Thermo Fisher cat#37515) the slides were incubated in antibody cocktail solution (Antibody Panel described in Supplementary file 1) overnight in 4^{0}C. Next day, the slides were washed in 0.2% Triton X-100 solution twice, TBS twice and incubated in Iridium DNA solution for 30 min in RT. The slides were air dried before setting up on the instrument. Imaging Mass cytometer was tuned before setting up the slides and the region of interest was drawn using H&E reference annotation. Images were processed using MCD Viewer software.

Macrophage differentiation and polarization

Peripheral blood leukocytes were isolated from healthy donor buffy coats (purchased from the New York blood bank) by centrifugation over a Ficoll–Paque Plus layer. CD14$^+$ cells were then isolated by magnetic sorting (positive selection). CD14$^+$ monocytes were differentiated into macrophages by culturing them for 5 days in complete medium (RPMI 10% FBS + Antibiotics) supplemented with 50 ng/mL of M-CSF or GM-CSF. At day 5, polarizing stimuli were added to induce the different canonical subsets: M1 (50 ng/mL IFNγ + LPS 1ng/ml), M2a (50 ng/mL IL-4), M2c (50 ng/mL IL-10 + 50 ng/mL TGF-β1), M2d (5 μM adenosine or 50ng/ml IL-6). PDTO-specific TAMs were induced by co-culturing them with PDTOs for 48hs at a 1:10 (macrophage: tumor cell) ratio.

Macrophage polarization assessment by bulk RNAseq

After macrophage polarization by co-culture with PDTOs, TAMs were recovered by magnetic sorting using MojoSort CD45$^+$ positive selection kit (cat #480168). Purified CD45$^+$ TAM cell pellets were submitted to the Weill Cornell Medicine Genomics Resources Core Facility for bulk RNA sequencing. Whole transcriptome library preparation and sequencing were performed using standard protocols on the Illumina NovaSeq platform. Bioinformatics analysis including quality control, alignment, and gene expression quantification was conducted by the core facility. DEG analysis was performed. For this manuscript, we only present the expression levels of selected genes representing conventional M1 markers (CD80, HLA-DRA, FCGR1A, IL1B, TNF), M2 markers (CD206, CD163, MERTK, IL10, TGFB1), and lung-associated TAM markers (PLAU, MARCO, TREM1, CHI3L1, CHI3L2, SPP1, APOE). Gene expression levels were normalized to M0 macrophage baseline expression and are reported as log2 fold change.

Macrophage phenotypic characterization by flow cytometry

Polarized macrophages were stained using a panel of conventional M1/M2 markers (MerTK, CD80, HLA-DR, CD163, CD206), lung-specific macrophages markers (TREM1, MARCO) and the differentially expressed transmembrane proteins on PDTOs-polarized TAMs (CD24, CD66c, CD66e).

Dimensionality reduction and clustering analysis: Flow cytometry data was analyzed using the Flowjo_V10 software employing Phenograph and FlowSOM plugins for clustering and tSNE for dimensionality reduction.

QUANTIFICATION AND STATISTICAL ANALYSIS

All statistical analyses were performed using GraphPad Prism (version 10.0, GraphPad Software, San Diego, CA). Data are presented as mean ± standard deviation (SD) or mean ± standard error of the mean (SEM) as indicated in figure legends. Statistical significance was set at $p < 0.05$ for all comparisons.

Associations between tumor size and PDTO/TIL establishment success rates were assessed using the Mann-Whitney test, while associations between disease stage and success rates were evaluated using the Chi-square test. Comparisons across multiple experimental conditions were performed using the Kruskal-Wallis test followed by Dunn's multiple comparisons post-test. Direct comparisons between two groups were analyzed using the Mann-Whitney test. Boxplots display median values with quartiles, while bar graphs show mean values with error bars representing SD or SEM as specified.

Cell Reports Methods, Volume *6*

Supplemental information

Tumor immune microenvironment reconstitution in patient-derived organoids enables therapy modeling for NSCLC

Enrique Podaza, Jared Capuano, Hui-Hsuan Kuo, Majd Al Assaad, Geoffrey Markowitz, M. Victoria Revuelta, John Nguyen, Adriana Irizarry, Hiranmayi Ravichandran, Sarah Ackermann, Troy Kane, Jyothi Manohar, Alyssa Duren-Lubanski, Michael Sigouros, Jenna Moyer, Bhavneet Bhinder, Pooja Chandra, Murtaza Malbari, Karsten Boehnke, Juan Miguel Mosquera, Vivek Mittal, Andrea Sboner, Hamza Gokozan, Nasser Altorki, Olivier Elemento, and M. Laura Martin

Supplemental information

Tumor immune microenvironment reconstitution in patient-derived organoids enables therapy modeling for NSCLC

Enrique Podaza, Jared Capuano, Hui-Hsuan Kuo, Majd Al Assaad, Geoffrey Markowitz, M. Victoria Revuelta, John Nguyen, Adriana Irizarry, Hiranmayi Ravichandran, Sarah Ackermann, Troy Kane, Jyothi Manohar, Alyssa Duren-Lubanski, Michael Sigouros, Jenna Moyer, Bhavneet Bhinder, Pooja Chandra, Murtaza Malbari, Karsten Boehnke, Juan Miguel Mosquera, Vivek Mittal, Andrea Sboner, Hamza Gokozan, Nasser Altorki, Olivier Elemento and M. Laura Martin

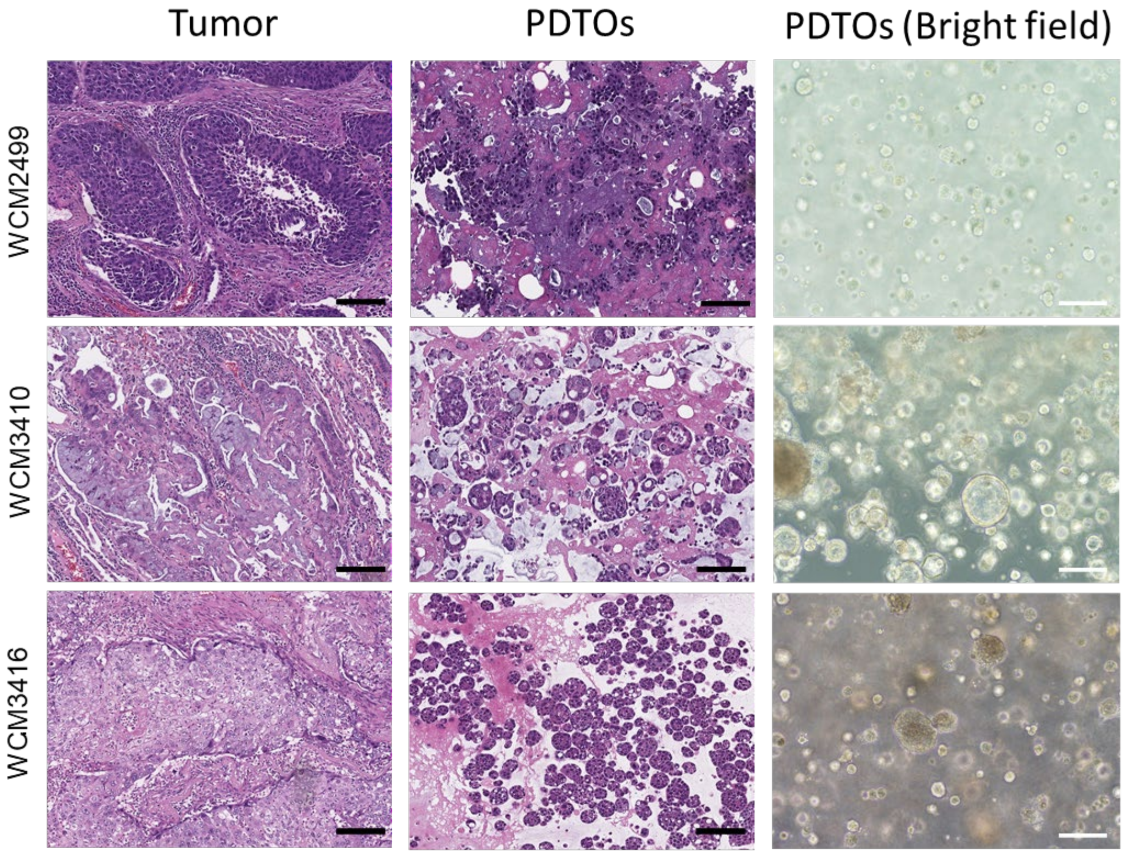

Figure S1. H&E of tumor and PDTO pairs for 3 cases of different subtypes of NSCLC: LCNEC (WCM2499), LUAD (WCM3410) and LUSC (WCM3416). Bright field images for the PDTOs are also displayed. Bars indicate 100µm. Related to Figure 1.

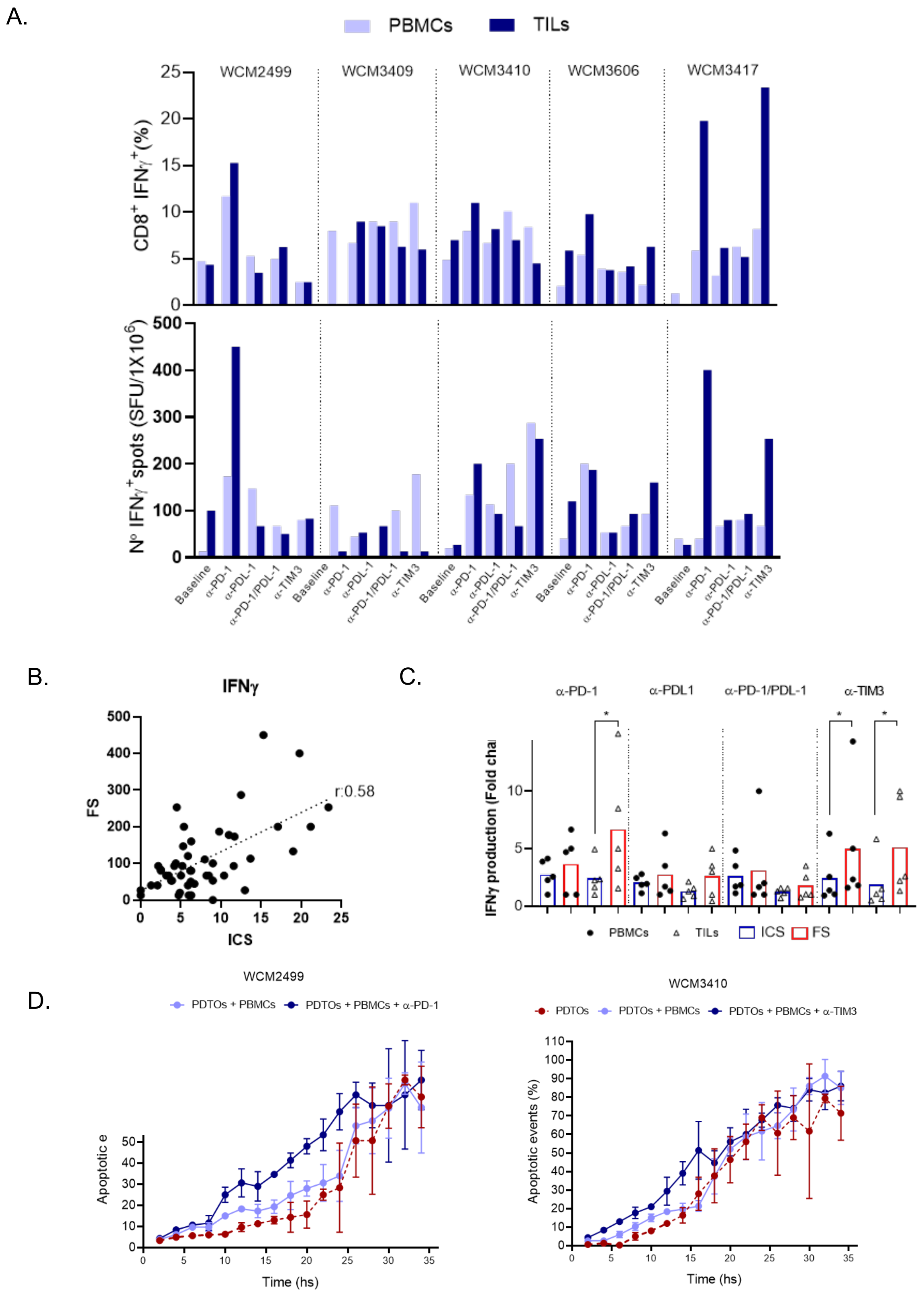

A.
PBMCs
TILs
WCM2499
WCM3409
WCM3410
WCM3606
WCM3417
CD8+ IFNγ+(%)
N° IFNγ+spots (SFU/1X10^6)
Baseline
α-PD-1
α-PDL-1
α-PD-1/PDL-1
α-TIM3
B.
IFNγ
FS
ICS
r:0.58
C.
α-PD-1
α-PDL1
α-PD-1/PDL-1
α-TIM3
IFNγ production (Fold cha
PBMCs
TILs
ICS
FS
D.
WCM2499
PDTOs + PBMCs
PDTOs + PBMCs + α-PD-1
Apoptotic e
Time (hs)
WCM3410
PDTOs
PDTOs + PBMCs
PDTOs + PBMCs + α-TIM3
Apoptotic events (%)
Time (hs)

Figure S2. Optimization of functional assays for the evaluation of T-cells IFNγ secretion and its modulation by immune-checkpoint inhibitors after co-culture with PDTOs.

A. INFγ secretion recorded by intracellular staining (ICS) and fluorospot (FS). Bars graphs display the frequencies of T-cells producing IFNγ, baseline and upon the addition of different mAbs. Frequencies are shown as percentage of $CD8^+$ $IFN\gamma^+$ for ICS (Upper graph) and as spots forming units (SFU) per 1 x 10^6 $CD8^+$ T-cells for FS (Lower graph). PBMCs and TILs frequencies for each patient are shown (n=5). **B.** Cross assay validation analysis. Correlation between IFNγ measures recorded by ICS and FS. Statistical significance was determined by Spearman's correlation (two tails, CI=95%). Spearman r coefficients are shown for each cytokine **C.** Comparison of IFNγ production by T-cells (TILs and PBMCs) in the presence of the different mAbs (α-PD-1, α-PDL1, α-PD-1/PDL1 and α-TIM3) recorded by ICS and FS. Values are shown as fold change relative to the control levels. (*) Statistical significance was calculated by Kruskal-wallis test, Dunn's multiple comparisons post-test (p=0.05). Black dots: PBMCs, White triangles: TILs, Blue bars: ICS, and Red bars: FS. n=5. **D.** Extended tumor killing assays**.** In this panel we show representative killing curves for two patients recorded up to 35 h. The curves show higher levels of dispersion at longer culture times. For this reason, 12 h of culture is shown on the main figures for all patients. Related to Figure 2.

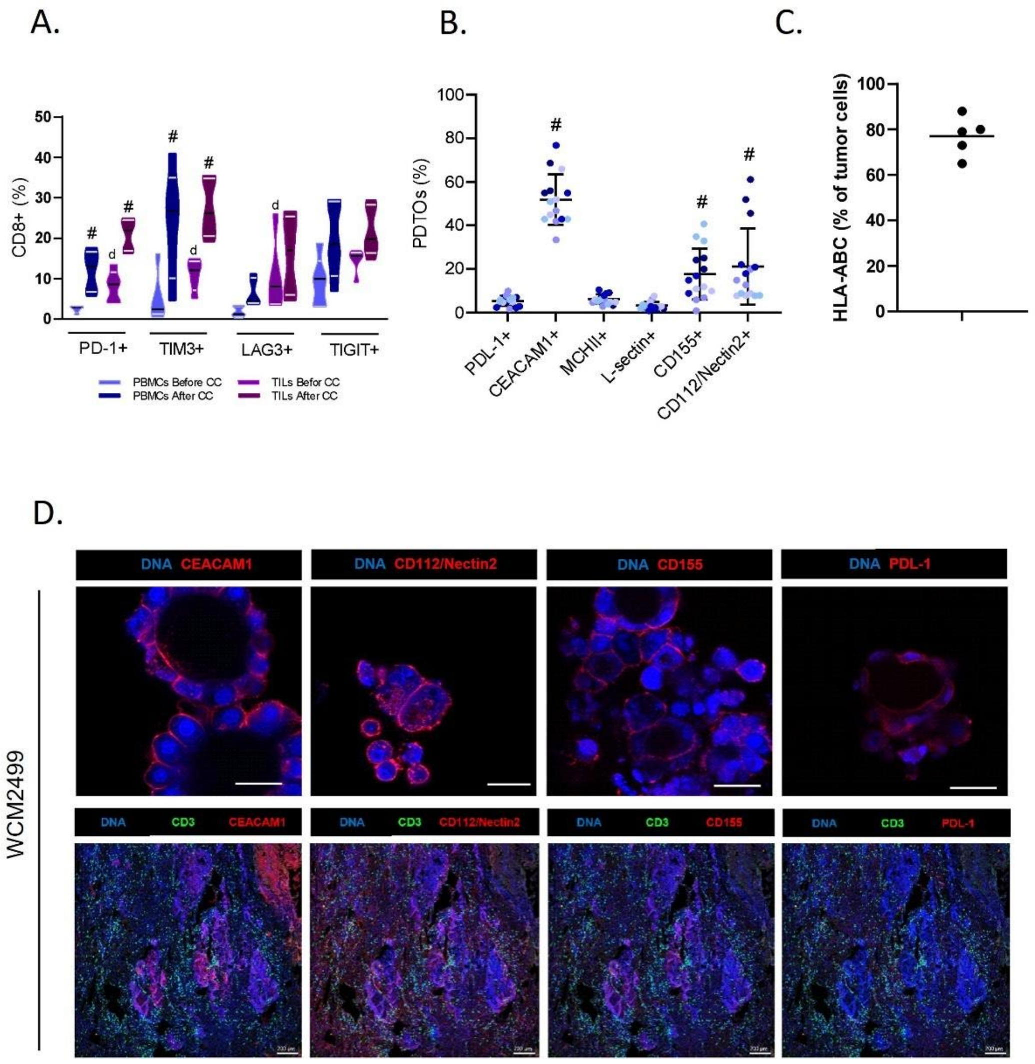

Figure S3. **Immune-checkpoints expression on T-cells and paired PDTOs**. **A.** Expression of inhibitory receptors Percentage of CD8+ cells expressing the inhibitory receptors PD-1, TIM3, LAG3 and TIGIT at day 0 and 14. Violin plots displaying the mean (black line) and quartiles (gray lines) for each inhibitory receptor in PBMCs and TILs before and after co-culture. # Indicates different than the condition before co-culture. d indicates significantly different than the PBMCs before co-culture. Differences were determined by Kruskal-Wallis test and Dunn's multiple comparison post-test (p=0.05) **B.** Percentage of PDTOs expressing PDL1, TIM3 ligand CEACAM, TIGIT ligands CD112/Nectin2, CD155, LAG3 ligands MCHII, and LSECtin. PDTO lines were stained 3 times at different passages between passage 6 and 15. # Indicate significantly higher than the PDL1 expression. Differences were determined by Kruskal-Wallis test and Dunn's multiple comparison post-test (p=0.05) **C.** Assessment of HLA-ABC expression on PDTOs before co-culture. Shown are the percentages of PDTOs positive for HLA-ABC**. D.** Representative confocal microscopy images of WCM2499 showing the surface expression of PDL1, CEACAM1, CD112/Nectin2, and CD155. Blue: DNA (DAPI); red: Immune checkpoint ligands (upper row). Imaging Cytoff of a tumor resection of WCM2499 showing the overall lymphocytic infiltration (CD3, green), DNA (blue) and expression of PDL1, CEACAM, CD112/Nectin2 and CD155 (red). Scale bars: 200μm (lower row). Related to Figure 3.

A.

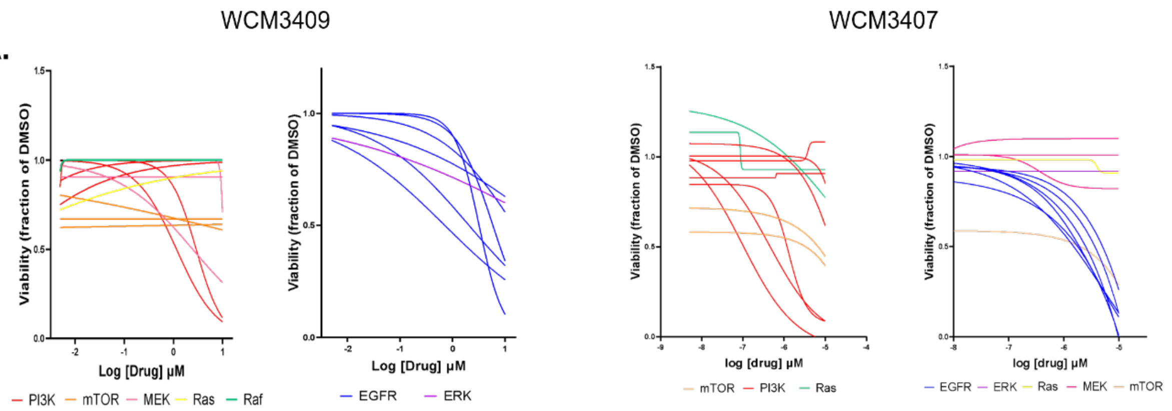

B.

WCM3409

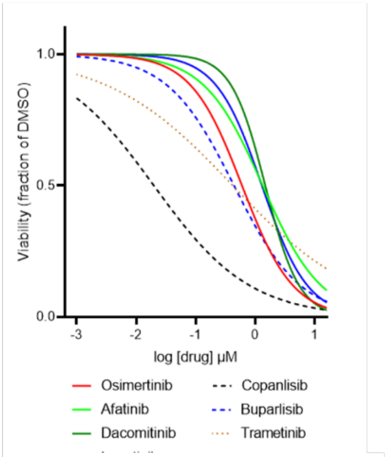

	EC10	EC20	EC25	EC50
**Afatinib**	0.268	0.5985	0.800	2.394
**Lapatinib**	0.431	0.47	0.9	1.877
**Osimertinib**	0.064	0.18	0.216	0.726
**Dacomitinib**	0.03	0.5	1.1	2.0
**Copanlisib**	0.005	0.016	0.021	0.064
**Buparlisib**	0.008	0.017	0.398	0.7655
**Trametinib**	0.015	0.032	0.08	0.136

C.

WCM3407

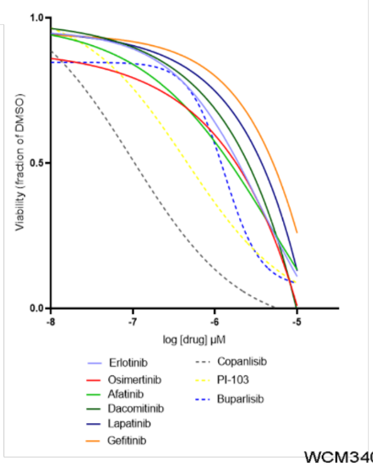

	EC10	EC20	EC25	EC50
**Afatinib**	5.85×10^{-7}	1.32×10^{-6}	1.32×10^{-6}	5.3×10^{-6}
**Lapatinib**	0.044	0.09	0.132	0.398
**Osimertinib**	4.13	9.3	12.4	37.20
**Dacomitinib**	0.07	0.162	0.216	0.6485
**Erlotinib**	5.85×10^{-7}	1.32×10^{-6}	1.32×10^{-6}	5.3×10^{-6}
**Gefitinib**	1.163×10^{-5}	2.62×10^{-5}	3.49×10^{-5}	0.0001047
**Copanlisib**	1.09×10^{-8}	2.46×10^{-8}	3.28×10^{-8}	9.86×10^{-8}
**Buparlisib**	1.47×10^{-7}	3.29×10^{-7}	4.39×10^{-7}	1.32×10^{-6}
**PI-103**	4.94×10^{-8}	1.11×10^{-7}	1.48×10^{-7}	4.44×10^{-7}

D.

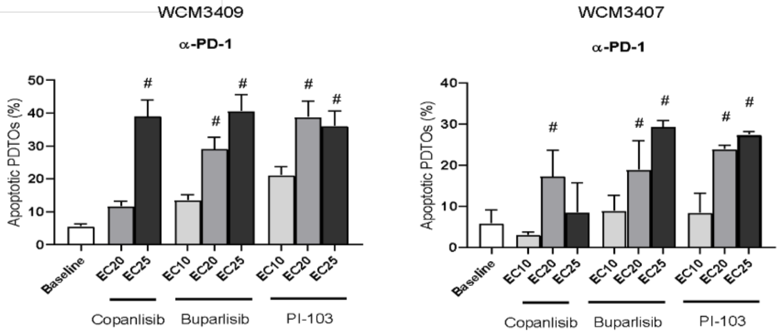

Figure S4. Kras G12A NSCLC-PDTOs sensitivity to target inhibitors.

PDTOs were digested into a single cell suspension and cells were plated in a 384 well plate at a density of 1000 cells per well in 8ul droplets (1:2 media:Matrigel). Plates were centrifuged briefly to ensure the cells were at the bottom of the well and 15µl of media were added. Cells were incubated for 72h to allow the cells to form PDTOs and afterwards the drugs were added and incubated for additional 96h. Concentrations ranging between 10 and 0 (serial 1/3 dilutions) were assessed for 23 different inhibitors against: EGFR (Lapatinib, Osimertinib, Dacomitinib, Afatinib, Erlotinib and Gefitinib), PI3K (Idelalisib, Copanlisib, PI-103, Buparlisib, GSK2636771, Parsaclisib and Erganelisib), mTOR (Temsirolimus, Rapamycin and Everolimus), ERK (Ulixertinib), Raf (AZ628 and Dabrafenib), Ras (AMF510) and MEK (Binimetinib,Trametinib and Selumetinib). The readout was performed using CellTiterGlo®3D reagent according to the manufacturer's protocol. Luminescence was measured by the Biotek Synergy H4 plate reader. Full dose-response curves are depicted for each patient in **A**. Each color represents a particular target. Dose response curves for the hit compounds and their estimated effective concentrations 10, 20, 25 and 50 are depicted for WCM3409 (**B**) and for WCM3407 (**C**). **D**. Apoptotic PDTOs (%) recorded for cells treated with α-PD-1 and PI3K inhibitors (Copanlisib, Buparlisib and PI-103). Mean + SD is displayed. The conditions depicted are those were the fold change between the ICI vs ICI + target inhibitor was greater than 2 and the observations across duplicates were consistent. Differences were determined by Kruskal-Wallis test and Dunn's multiple comparison post-test (p=0.05). # means significantly different than baseline. Related to Figure 3.

A.

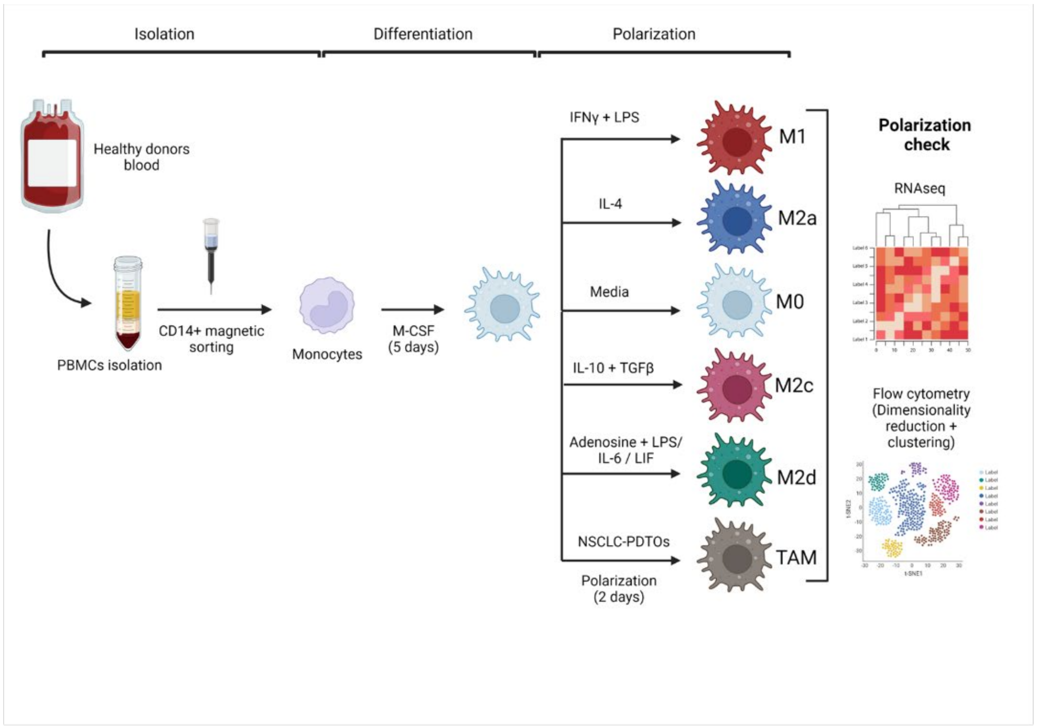

B.

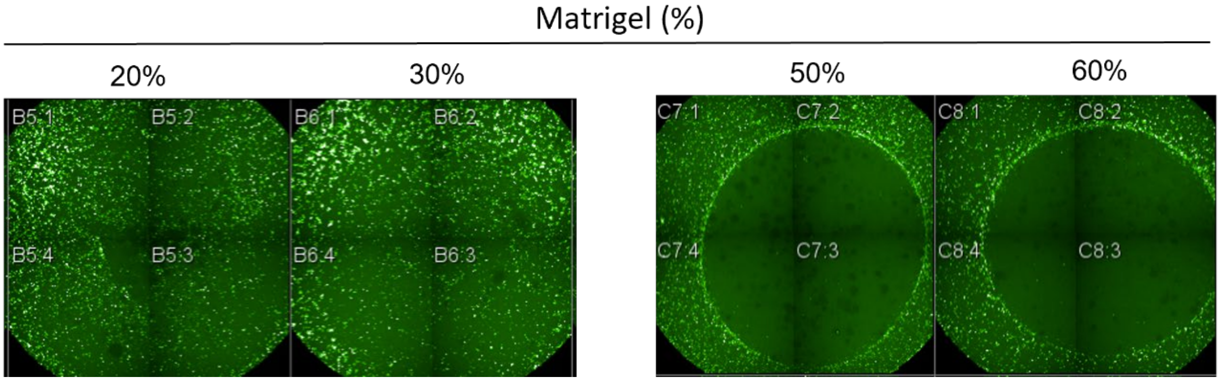

Figure S5. Macrophage generation and characterization workflow.
A. Macrophage differentiation and polarization protocol. FBMCs were isolated from healthy donor leukopacks. Monocytes were purifed from PBMCs suspensions by magneto sorting (CD14 positive selecton). Purifed monocytes were differentiated into macrophages by culturing them in the presence of M-CS during 5 days. MO macrophages were polarized to the different subsets by culturing them during 48hs with subset specifc factors. For the particular case of TAMs polarization, MO macrophages were oo cultured with tumor oells in a 1:10 ratio (MO: tumor oell). After oo-culture, CD45+ magne to sorting was used to eliminate tumor oells. Polarized macrophages were characterized by RNAseq (followed by qPCR validation of selected genes) and flow cytometry employing conventional M1/M2 markers as well as Lung-associated macrophages markers. B. Matrigel concentration assessment. CFSE-stained macrophages were co-culture with PDTOs employing different Matrigel concentrations (20, 30, 50 and 80%) and images were acquired at day 12 of culture. Related to Figure 4.

ID	TILs
WCM1327	Fail
WCM1335	Fail
WCM1332	Fail
WCM1341	Fail
WCM1518	Succeed
WCM1532	Fail
WCM1536	Succeed
WCM1548	Succeed
WCM1547	Fail
WCM1471	Fail
WCM1478	Fail
WCM1442	Fail
WCM1412	Fail
WCM1445	Fail

Table S1.TILs expansion outcome for tumor samples digested with collagenase IV. Related to Figure 1.

Patient	Gender	Race/ Ethnicity	Smoking status	Cancer stage	Lesion size on pathologic examination (cm)	T stage	N stage	M stage
**WCM3407**	M	White	Former	IIB	4.0	T3	N0	M0
**WCM2499**	F	White	Former	IIIA	7,6	T4	N0	M0
**WCM3606**	F	African American	Former	IIA	3,8	T2a	N1	M0
**WCM3417**	F	White	Never	IA3	3	T1c	N0	M0
**WCM3409**	F	African American	Never	IA3	2,9	T1c	N0	M0
**WCM3410**	F	White	Former	IIIA	7,3	T4	N0	M0
**WCM3602**	M	White	Current	IIIA	3,3	T2a	N2	M0
**WCM3603**	F	White	Never	IA2	1,7	T1b	N0	M0
**WCM3604**	M	White	Never	IB	3,3	T2a	N0	M0
**WCM3605**	F	White	Never	IA3	2,6	T1c	N0	M0
**WCM3413**	M	White	Former	IIIA	3	T1b	N0	M0
**WCM3066**	M	White	Current	IA2	1,9	T3	N1	M1a
**WCM3607**	F	White	Never	IA3	2,1	T1c	N0	M0
**WCM3416**	M	White	Former	IIB	5,5	T3	N0	M0
**WCM3608**	M	Asian	Never	IB	3,5	T2a	N0	M0
**WCM3083**	F	unknown	Current	IVA	4	T3	N1	M1a
**WCM3289**	M	Asian	Former	IIIB	6,2	T3	N2	M0

Table S2. Patients clinical information. Related to Figure 1

ID	TILs	Histopathological review
WCM3407	No	Scattered infiltration. Presence of macrophages
WCM2499	Yes	High lymphocytic infiltration in tumor stroma
WCM3606	Yes	No infiltration. T-cells present in surrounding normal tissue
WCM3417	Yes	No infiltration. T-cells present in surrounding normal tissue
WCM3409	Yes	Moderate and scattered infiltration
WCM3410	Yes	High Lymphocytic infiltration in tumor stroma
WCM3413	No	Scattered infiltration
WCM3416	No	Scattered infiltration. Presence of macrophages
WCM3289	Yes	No infiltration. T-cells present in surrounding normal tissue

Table S3- Tumor immune infiltration histopathological report and TILs culture success (Yes-Succeed, no: Fail).
Related to Figure 1.
